# Beyond n-dopants for organic semiconductors: use of bibenzo[*d*]imidazoles in UV-promoted dehalogenation reactions of organic halides

**DOI:** 10.3762/bjoc.19.142

**Published:** 2023-12-14

**Authors:** Kan Tang, Megan R Brown, Chad Risko, Melissa K Gish, Garry Rumbles, Phuc H Pham, Oana R Luca, Stephen Barlow, Seth R Marder

**Affiliations:** 1 Renewable and Sustainable Energy Institute (RASEI), University of Colorado Boulder, Boulder, Colorado 80309, United States; 2 Department of Chemistry & Center for Applied Energy Research (CAER), University of Kentucky, Lexington, Kentucky, 40506, United States; 3 National Renewable Energy Laboratory, Chemistry and Nanoscience Center, Golden, Colorado, 80401, United States; 4 Department of Chemical and Biological Engineering, University of Colorado Boulder, Boulder, Colorado 80309, United States; 5 Department of Chemistry, University of Colorado Boulder, Boulder, Colorado 80309, United States

**Keywords:** dehalogenation, n-dopant, reduction, reductive dimerization

## Abstract

2,2’-Bis(4-dimethylaminophenyl)- and 2,2'-dicyclohexyl-1,1',3,3'-tetramethyl-2,2',3,3'-tetrahydro-2,2'-bibenzo[*d*]imidazole ((N-DMBI)_2_ and (Cyc-DMBI)_2_) are quite strong reductants with effective potentials of ca. −2 V vs ferrocenium/ferrocene, yet are relatively stable to air due to the coupling of redox and bond-breaking processes. Here, we examine their use in accomplishing electron transfer-induced bond-cleavage reactions, specifically dehalogenations. The dimers reduce halides that have reduction potentials less cathodic than ca. −2 V vs ferrocenium/ferrocene, especially under UV photoexcitation (using a 365 nm LED). In the case of benzyl halides, the products are bibenzyl derivatives, whereas aryl halides are reduced to the corresponding arenes. The potentials of the halides that can be reduced in this way, quantum-chemical calculations, and steady-state and transient absorption spectroscopy suggest that UV irradiation accelerates the reactions via cleavage of the dimers to the corresponding radical monomers.

## Introduction

**Figure 1:**
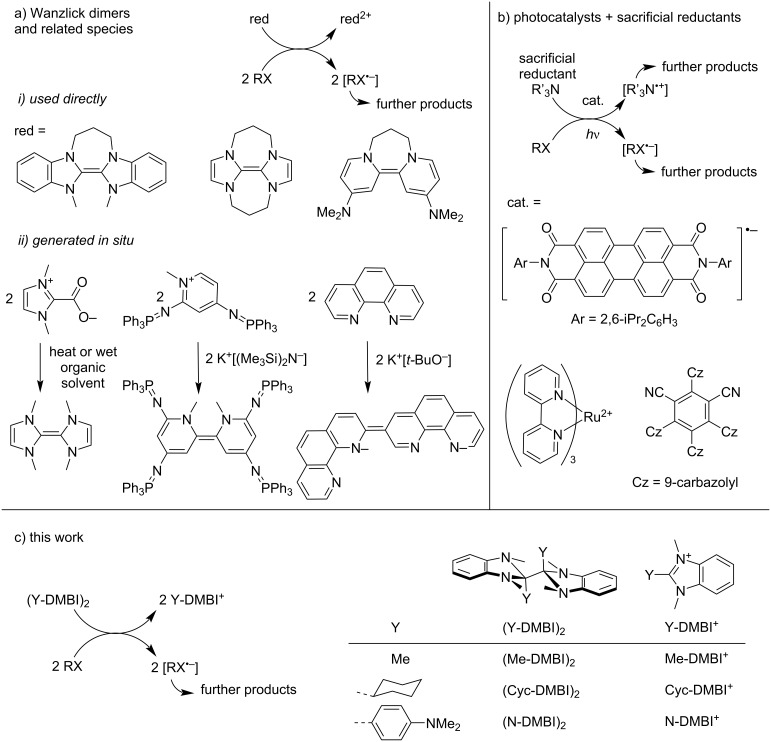
(a, b) Schematics of previous approaches to dehalogenation-based reactions using molecular reductants, along with representative structures of reductants and photocatalysts used, and (c) schematic for the present approach, along with structures of 1,1',3,3'-tetramethyl-2,2',3,3'-tetrahydro-2,2'-bibenzo[*d*]imidazoles, (Y-DMBI)_2_, and the corresponding monomer cations, specifically the first reported in the literature and those used in this work.

Reductive dehalogenation reactions of organic halides can be used in organic synthesis as a means of generating carbon-centered radical or anion intermediates and could have relevance to the treatment of waste halogenated polymers. While such reactions can be achieved using highly reducing metals, molecular reductants can potentially enable more selectivity, as required for the use of such reactions in the synthesis of molecules bearing various functional groups. In particular, Wanzlick dimers (C=C-bonded dimers of N-heterocyclic carbenes, Figure 1a, i) have been used by Murphy’s group and others for a variety of transformations, such as the formation of indolines from *N*-allyl-2-iodoanilines [1], indanones from 3-(2-halophenyl)propanoic esters [2–3], and 3-methyl-2,3-dihydrobenzofuran from 1-allyloxy-2-halobenzenes [4]. Related species have also been used to initiate the coupling of aryl halides and arenes [5]. However, even relatively easily reduced organic halides have sufficiently cathodic reduction potentials (e.g., ca. −1.6 V and −1.8 vs ferrocenium/ferrocene (FeCp_2_^+/0^) for diethyl bromomalonate [6] and 4-iodotoluene, see Table 2, respectively) that simple one- or two-electron donors capable of exergonic ground-state electron transfer to these substrates will be rather air sensitive, complicating their handling and use. In addition some molecular reductants can themselves react with the reactive intermediates; for example, the dehalogenation of alkyl halides, RX, by CoCp_2_ (Cp = η^5^-cyclopentadienyl; *E* = −1.3 V vs FeCp_2_^+/0^), gives CoCp_2_^+^ and X^−^, but the organic radicals R^•^ react with another molecule of CoCp_2_ to afford CoCp(η^4^-C_5_H_5_R) [7]. In some cases, issues of reductant air sensitivity can be circumvented by the use of photocatalysts in concert with sacrificial weak reductants (Figure 1b) [8–11]. Another approach is to add ambient-stable precursors to reaction mixtures: for example, reducing Wanzlick dimers and related species (Figure 1a, ii) have been formed from precursors through in situ decarboxylation [12] or deprotonation [4–5], while other reducing species have been formed from in situ reactions of simple diols or diamines [13].

Another approach is to utilize dimers formed by highly reducing radicals, such the bibenzoimidazoles (Y-DMBI)_2_ (Figure 1c). (Me-DMBI)_2_ was first reported in 1984 and used as a reductant in studies of electrochemically generated reactive species [14–16]. More recently, several examples, including (Cyc-DMBI)_2_ (Y = cyclohexyl) and (N-DMBI)_2_ (Y = 4-dimethylaminophenyl) have been used as effective n-dopants for organic semiconductors [17–24] and redox mediators for the electrochemical depolymerization of poly(ethylene terephthalate) [25]. These dimers (D_2_ = (Y-DMBI)_2_) undergo reactions with organic semiconductors A to afford two monomeric Y-DMBI^+^ (D^+^) cations and two reduced semiconductors, A^•−^. The effective redox potentials, *E*(D^+^/0.5D_2_), are estimated to be ca. −2 V vs FeCp_2_^+/0^, yet the dimers are reasonably stable to air due to the kinetic barriers associated with the coupling of electron-transfer and bond-cleavage reactions [26]. Here we demonstrate that (N-DMBI)_2_ and (Cyc-DMBI)_2_ (Figure 1c) can be used to accomplish dehalogenation of benzyl, alkyl, and aryl halides (RX) and discuss the scope and possible mechanism of these reactions.

## Results and Discussion

### Reaction of (Y-DMBI)_2_ with benzyl bromide

We began our investigations of dehalogenation reactions using benzyl bromide (BnBr, **1a**), which has a reduction peak potential (*E*_pc_) of −1.6 V vs FeCp_2_^+/0^, as the substrate (RX), anhydrous THF (without stabilizer) as the solvent, and (N-DMBI)_2_ or (Cyc-DMBI)_2_ as a stoichiometric reductant (0.5 equiv, assuming one electron is needed for each substrate molecule and that each dimer molecule contributes two electrons). Reaction outcomes were analyzed by GC–MS (see Supporting Information File 1 for details); products were identified based on the observed *m*/*z* values and comparison of retention times with authentic samples, while conversions and yields were based on calibration curves established using authentic samples. Table 1 summarizes the conversion of BnBr and product yields at various reaction times, with or without dimers, and with or without irradiation at a nominal wavelength of 365 nm (see Supporting Information File 1 for experimental details and see [27] for the spectrum of the excitation source used), which is anticipated to selectively excite the dimeric reductants rather than the substrate (see below and Supporting Information File 1, Figure S4 for further details). In the dark at low concentrations of substrate and reductant low conversions are observed after 1 h (Table 1, entry 1), with the dehalogenation products being a mixture of toluene and bibenzyl, i.e., RH and R_2_ products, which were identified by the masses observed through GC–MS and through comparison of the GC–MS retention times with authentic samples. Higher concentrations and reaction times (entries 7–9 in Tabe 1) lead to larger extents of conversion (up to 61% at 18 h) and favor formation of bibenzyl over toluene (up to 52% yield at 18 h). [BnBr]^•–^ presumably cleaves to afford Bn^•^, which can react further to form Bn_2_ (see following section) or can form toluene through reaction with THF, which is known to have a reasonably weak α-CH bond and act as a H^•^ donor towards many radicals [28–31].

**Table 1:** Debromination of benzyl bromide (**1a**).

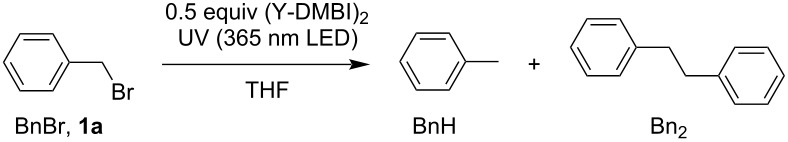							

Entry	Time [h]	UV	[BnBr] [mM]^a^	Reductant	Conversion [%]^b^	BnH yield [%]^b^	Bn_2_ yield [%]^b^

1	1	no	3.0	(N-DMBI)_2_	20	13	10
2	0.5	yes	3.0	(N-DMBI)_2_	90	8.6	82
3	1	yes	3.0	(N-DMBI)_2_	100	9	92
4	1	no	3.0	(Cyc-DMBI)_2_	10	5	5
5	0.5	yes	3.0	(Cyc-DMBI)_2_	70	4	65
6	1	yes	3.0	(Cyc-DMBI)_2_	100	4	95
7	1	no	18.7	(N-DMBI)_2_	29	7.5	22
8	2	no	18.7	(N-DMBI)_2_	39	9	30
9	18	no	18.7	(N-DMBI)_2_	61	7	52
10	1	yes	18.7	(N-DMBI)_2_	70	6	66
11	2	yes	18.7	(N-DMBI)_2_	80	6	74
12	2	yes	18.7	–	6	6	–^c^
13	4	yes	18.7	(N-DMBI)_2_	92	6	86
14	18	yes	18.7	(N-DMBI)_2_	100	8	93
15	18	yes	18.7	–	61	58	–^c^

^a^3.0 and 18.7 mM BnBr concentrations used, BnBr in quantities of 12 and 75 µmol (2 and 13 mg), respectively, corresponding to dimer quantities of 6.0 and 37.5 µmol, respectively (3.2 and 20 mg for (N-DMBI)_2_; 2.8 and 17 mg for (Cyc-DMBI)_2_. ^b^Conversions and yields were determined by GC–MS as described in Supporting Information File 1. ^c^None detected.

The reaction is substantially accelerated by UV excitation at 365 nm; quantitative conversion of benzyl bromide at low initial concentration (3 mM) can be achieved within 1 h using both (N-DMBI)_2_ or (Cyc-DMBI)_2_ and UV (Table 1, entries 3 and 6), while even at higher substrate concentrations (18.7 mM) 80% and near-quantitative (>90%) conversions can be obtained using (N-DMBI)_2_ within 2 h and 4 h, respectively (Table 1, entries 11 and 13). We also investigated the impact of photoexcitation in the absence of the dimeric reductant; however, extents of conversion are much lower for a given reaction time (compare entries 12 and 15 to 11 and 14 in Table 1) and the sole detected product is toluene rather than bibenzyl.

Furthermore, in one of the cases of complete conversion (Table 1, entry 14), ^1^H NMR spectroscopy indicated that the reductant-based side product is a salt of the monomeric cation N-DMBI^+^ (Supporting Information File 1, Figure S15). Thus, the overall reaction is consistent with:


[1]

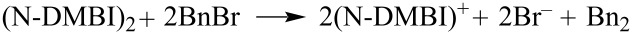



### Scope of reaction (Y-DMBI)_2_ with other benzyl, alkyl, and aryl halides

Table 2 summarizes the conversions and product yields for the reactions of (N-DMBI)_2_ or (Cyc-DMBI)_2_ with several other benzyl halides (**1b**–**e**), an alkyl halide **2**, and several aryl halides (**3a**–**f**). Again GC–MS was used to identify and quantify the products; the necessary authentic samples were mostly commercially available, but the R_2_ products from **1b** and **1c** were not, although well-known in the literature (for example, see ref. [32]), and were synthesized as described in Supporting Information File 1. More complete data are shown in Supporting Information File 1, Tables S1 and S2. As in the case of **1a**, conversions and yields under UV irradiation in the absence of reductant are low on a 2 h timescale (≤10%) and the main products are those in which the halide is replaced by a hydrogen atom. The more easily reduced benzyl halides examined (**1b** and **1c**) are dehalogenated by (N-DMBI)_2_ in the dark, and with (N-DMBI)_2_ and UV irradiation are quantitatively dehalogenated in 2–6 h with the corresponding substituted bibenzyls being the dominant products. These reaction conditions represent an alternative metal-free approach to the conventional synthesis of bibenzyls through the reaction of Grignard or organolithium reagents with benzyl halides, or to the use of highly active metal reagents [33–36] or metal-containing catalysts [32]. We note that another all-organic reductive dimerization of benzyl halides using 2,3,5,6-tetrakis(tetramethylguanidino)pyridine has recently been reported [37]. The less readily reduced halides examined here (**1d**,**e**, and **2**) are only sluggishly converted, even when using both (N-DMBI)_2_ (or (Cyc-DMBI)_2_) and light. Moreover, in the cases of **1d** and **1e** there are significant mismatches between conversion and the yields of the corresponding RH and R_2_ species, indicating that additional products must be formed. Indeed, in the case of 4-methylbenzyl chloride GC–MS shows a product with a mass consistent with the formation of 4-methylbenzyl-substituted THF (see Supporting Information File 1, Figure S2) and a somewhat better R_2_ yield is obtained in toluene (see Table S2). For 1-bromooctadecane (**2**), around half the product obtained using (N-DMBI)_2_ and UV is RH*,* i.e., octadecane. The remaining unidentified product may be R_2_ (C_36_H_74_), but this product is not easily detected by GC–MS.

**Table 2:** Dehalogenation of various benzyl, alkyl, and aryl halides using (N-DMBI)_2_ (or, in parentheses Cyc-DMBI)_2_.^a^

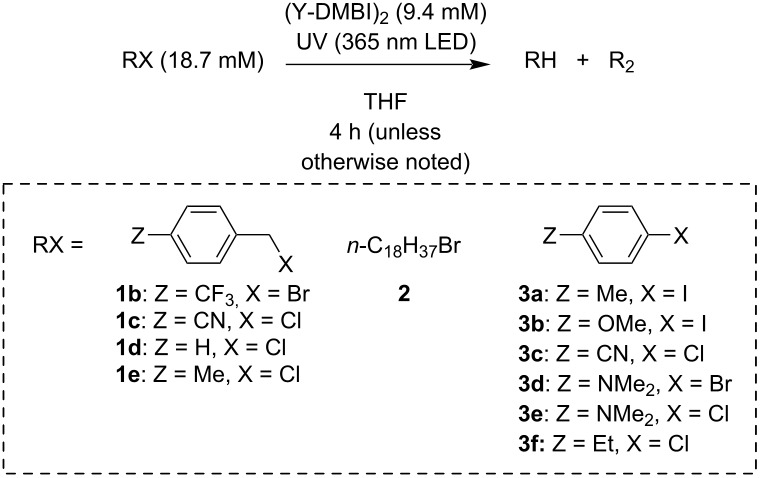				

RX	*E*_pc_ [V]^b^	Conversion [%]^c^	RH yield [%]^c^	R_2_ yield [%]^c^

**1a**	−1.6	92	6	86
**1b**	−1.5	80	2	75
**1c**	−1.6	100	14	86
**1d**	−2.3	39	7	5
**1e**	−2.4	73 (60)	3 (3)	3 (32)
**2**	−2.1	39	19	–^d^
**3a** ^e^	−1.8	98	98	–^f^
**3b** ^e^	−1.6	99	99	–^f^
**3c**	−1.6	55 (99)	41 (60)	–^f^ (–^f^)
**3d**	−2.4	5 (5)	5 (5)	–^f^
**3e**	−2.5	4 (2)	0 (2)	–^f^
**3f**	−2.4	1 (<1)	1 (<1)	–^f^

^a^The reaction scale in each case was 75 µmol (9.5–25 mg) RX and 37.5 µmol dimer (20 and 17 mg for (N-DMBI)_2_ and (Cyc-DMBI)_2_, respectively). ^b^Peak reduction potential vs FeCp_2_^+/0^ in THF/0.1 M Bu_4_NPF_6_ (see Supporting Information File 1, Figures S7 and S8). ^c^Conversions and yields were determined by GC–MS as described in Supporting Information File 1. ^d^Likely not detectable by GC–MS. ^e^2 h reaction time. ^f^None detected.

In sharp contrast to the case of benzyl derivatives, where the use of dimer reductants primarily affords R_2_ products, biaryls are not observed as dehalogenation products of aryl halides by dimeric reductants and/or light. This may be attributable to the lower stability and greater reactivity of aryl radicals relative to that of their benzyl counterparts. Indeed, aryl radicals are known to abstract H^•^ from THF [30–31] and presumably do so in the present reactions before any further reactions can occur. Resonance-stabilized benzyl radicals, on the other hand, are sufficiently long-lived to react further to afford dimeric bibenzyl derivatives (especially at higher concentrations or when photoirradiation is used, presumably affording higher steady-state [R^•^] concentrations). In principle, this is possible by either dimerization of 2R^•^ or by a second reduction of R^•^ to R^−^ (as invoked in the reductive cyclization of (2-halophenyl)propanoic esters [2]) which then acts as a nucleophile towards a second molecule of RX. However, addition of Me_3_SiCl to a photoirradiated BnBr/(N-DMBI)_2_ reaction mixture did not lead to any detectable BnSiMe_3_, thus supporting a radical dimerization pathway (see Supporting Information File 1, Table S3).

As in the case of sp^2^ R–X systems, only small extents of dehalogenation for R = aryl are observed in the absence of reductants. For the more easily reduced aryl halides (**3a**–**c**), moderate to high extents of conversion are obtained in the dark using dimeric reductants, while higher extents, in some cases near-quantitative in 2 h, are obtained using UV and reductants. In the case of **3a** and **3b** the conversion and yields of the RH compounds are in good agreement, while discrepancies in the case of **3c** indicate additional side reactions.

**Scheme 1:**
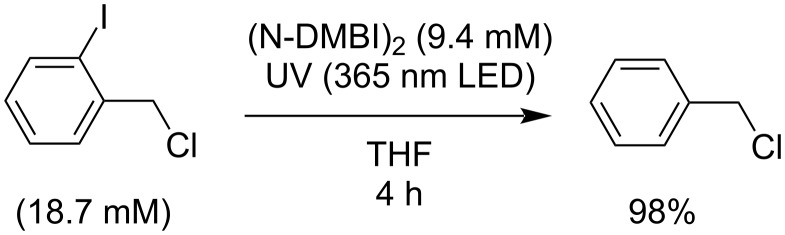
Selective deiodination of 2-iodobenzyl chloride.

The observation that some of the organic halides tested in Table 2 are cleanly dehalogenated and others essentially unreactive suggested the possibility of selective dehalogenation of compounds containing different halogenated functionalities. Specifically, we examined 2-iodobenzyl chloride, in which the C–I and C–Cl bond strengths are expected to be fairly similar (C–X bond dissociation enthalpies of 280 and 310 kJ mol^−1^ have been reported for BnCl and PhI, respectively [38]), but the aryl–I bond is likely to be better coupled to the reduction process (given the reduction potentials in Table 2). As shown in Scheme 1, we found 2-iodobenzyl chloride is cleanly converted to benzyl chloride under the standard conditions used in Table 2 (see Supporting Information File 1, Table S4 for more details). This selectivity is not typically achievable using electropositive metals and may be of use in more elaborate chemical transformations.

### Mechanism of dark reactions

**Scheme 2:**
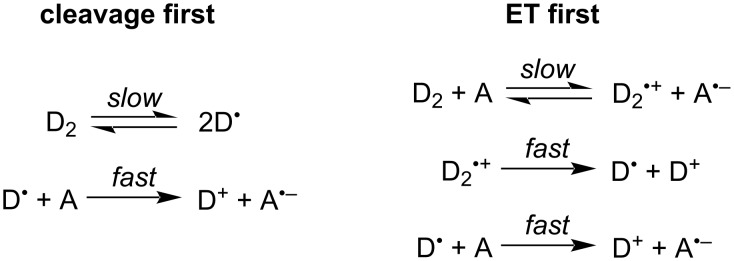
Reaction mechanisms for the reactions of dimeric reductants (D_2_) such as (Y-DMBI)_2_ derivatives with acceptors (A) such as organic semiconductors or, in this work, organic halides that react further (the relative rates of steps are indicated for cases where the A reduction potential falls between D_2_ and D^•^ oxidation potentials).

Doping of organic semiconductors by (Y-DMBI)_2_ dimers [18,39] or by various dimers formed by 18-electron sandwich compounds [18,40–41], as well as redox reactions of other dimers formed by organic radicals [42–43], are known to proceed by two distinct pathways. When the dimer (D_2_) is relatively weakly bound, its dissociation to D^•^ can be the initial step (“cleavage-first”), which is then followed by fast electron-transfer (ET) reactions (Scheme 2). On the other hand, in the “electron-transfer first” mechanism, the first step is an ET reaction, resulting in the formation of D_2_^•+^, which subsequently cleaves to form D^+^ and D^•^, which is much more readily oxidized than the dimer itself and thus participates in a second fast ET (Scheme 2). In the present case, knowledge of the operative mechanism(s) is important to understand what substrates might be cleavable on what timescales in the dark; in particular, if the cleavage-first mechanism is viable, substrates with *E*_red_ as cathodic as, roughly, *E*(D^+^/D^•^), should react, whereas more strongly bonded dimers might not react with the most challenging of these substrates due to the non-viability of the cleavage-first mechanism and prohibitively endergonic initial steps for the ET-first mechanism.

**Figure 2:**
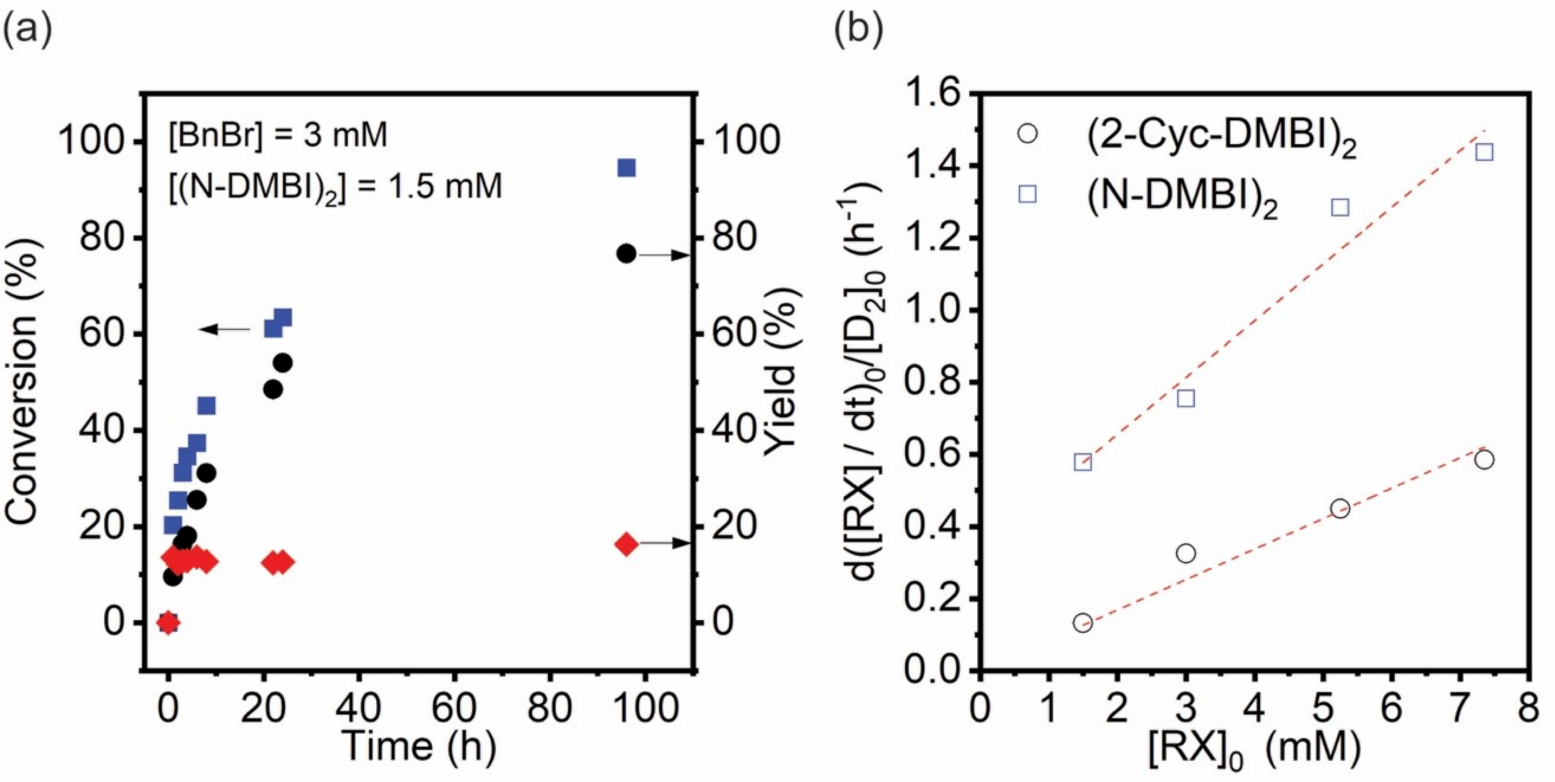
(a) A representative temporal evolution of % conversion (blue squares), % toluene yield (red diamonds), and % bibenzyl yield (black circles) during the dark dehalogenation reaction of benzyl bromide (BnBr) using (N-DMBI)_2_ in THF (these data were acquired using 3 mM BnBr and 1.5 mM of (N-DMBI)_2_. (b) Plot of [D_2_]_0_-normalized initial reaction rate (d([RX]/d*t*)_0_/[D_2_]_0_) as a function of the initial benzyl bromide concentration ([RX]_0_) obtained from several experiments of the type shown in part (a) for different [D_2_] and [RX] for both D_2_ = (Cyc-DMBI)_2_ and (N-DMBI)_2_. For these slow reactions, the “initial” rates were estimated from the change of substrate concentration over the first 30 min reaction time.

We investigated the dark reactions of the two dimers with BnBr in THF, using GC–MS to determine RX conversion and R_2_ and RH yields at various time intervals, as shown in the example of Figure 2a. We repeated these experiments for a variety of different initial reductant and RX concentrations, [D_2_]_0_ and [RX]_0_ (see Supporting Information File 1, Figure S3), and plotted the initial rate normalized for dimer concentrations, (d[RX]/d*t*)_0_/[D_2_]_0_, vs [RX]_0_ (Figure 2b). In the case of D_2_ = (Cyc-DMBI)_2_, a linear plot with approximately zero intercept is obtained, consistent with a reaction first order in [RX] and [D_2_], as expected if the reaction proceeds via an initial rate-determining ET. On the other hand, in the case of D_2_ = (N-DMBI)_2_, the linear plot has a distinctly non-zero intercept, a behavior which conforms with a rate law consisting of the sum of two terms, one first order in [D_2_] and one first order in both [D_2_] and [RX]


[2]

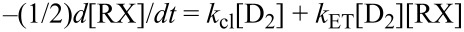



and is similar to doping behavior we have recently seen for some weakly bonded dimers where both “cleavage-first” and “ET-first” mechanisms are competitive. In the case of (Cyc-DMBI)_2_, the second-order rate constant, *k*_ET_, is estimated as 6.0 × 10^−3^ M^−1^ s^−1^, whereas for (N-DMBI)_2_, *k*_cl_ = 4.7 × 10^−5^ s^−1^ and *k*_ET_ = 1.0 × 10^−2^ M^−1^s^−1^. The difference in *k*_ET_ values is qualitatively consistent with the peak oxidation potentials, *E*_pa_(D_2_^•+^/D_2_), of the two dimers; values of −0.06 and −0.13 V vs FeCp_2_^+/0^ are found for (Cyc-DMBI)_2_ and (N-DMBI)_2_, respectively [18,44], indicating that ET from the former to benzyl bromide is more endergonic than from the latter. The rate constant for (Cyc-DMBI)_2_-to-BnBr ET is also much smaller than that previously determined for the doping of 6,13-bis(triisopropylsilylethynyl)pentacene (TIPS-pentacene, *E*_1/2_^0/−^ = −1.45 V, *k*_ET_ ≈ 0.15 M^−1^s^−1^) by (Cyc-DMBI)_2_ [18], consistent with the differences in the reduction potentials between BnBr and TIPS-pentacene. The observation of the “cleavage-first” mechanism for (N-DMBI)_2_ and not for (Cyc-DMBI)_2_ is consistent with DFT estimates of bond dissociation energies for these two dimers (Δ*U*_diss_ = 163 and 210 kJ mol^−1^, respectively [18,44]) and with their reactivity towards TIPS-pentacene [18,39].

### Impact of photoexcitation

**Figure 3:**
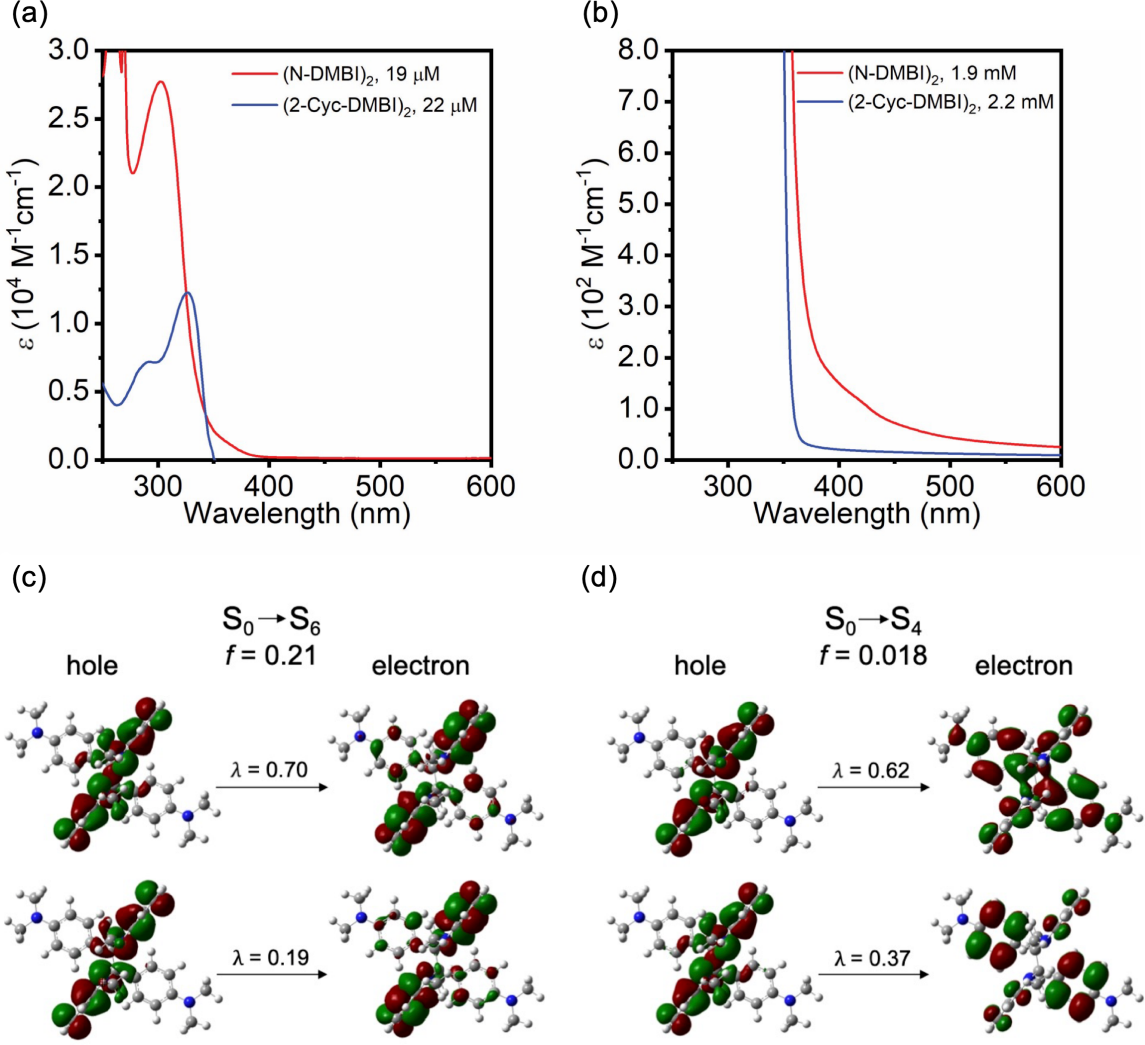
Top: UV–vis absorption spectra for the two dimeric reductants in THF emphasizing (a) the different positions of the relatively strong absorption peaks and (b) the presence of a low-energy shoulder in the spectrum of (N-DMBI)_2_. Bottom: TD-DFT (M06/6-31G(d, p)) natural transition orbitals for selected (N-DMBI)_2_ transitions that are thought to be primarily responsible for (c) the main absorption peak and (d) the weak shoulder seen experimentally.

The absorptivities, ε, of (N-DMBI)_2_ and (Cyc-DMBI)_2_ at 365 nm in THF are ca. 420 and 43 M^−1^ cm^−1^, respectively. Figure 3a and b show the absorption spectra of the two dimeric reductants in THF (see Supporting Information File 1, Figure S6 for data in toluene). (N-DMBI)_2_ shows a strong absorption feature with a maximum at 304 nm (ε_max_ = 28000 M^−1^ cm^−1^) and a weak shoulder at ca. 400 nm (ε_400_ ≈ 150 M^−1^ cm^−1^), whereas (Cyc-DMBI)_2_ exhibits only a strong feature with a maximum at 327 nm (ε_max_ = 13000 M^−1^ cm^−1^). TD-DFT calculations (M06/6-31G(d,p), isolated molecules) qualitatively reproduce the different behavior of the two dimers: for (Cyc-DMBI)_2_ the S_0_→S_1_ transition is calculated to be strong (oscillator strength, *f* = 0.27). The natural transition orbitals (NTOs, see Supporting Information File 1, including Figure S10, for more information) indicate that this excitation is largely confined to the bibenzoimidazole portion of the molecule. In the case of (N-DMBI)_2_ the lowest reasonably strong (*f* = 0.21) transition is S_0_→S_6_ and has bibenzoimidazole-dominated NTOs (Figure 3c) similar to those for the S_0_→S_1_ transition of (Cyc-DMBI)_2_, but is seen at higher energy. The strongest of the weaker lower energy transitions are S_0_→S_4_ (*f* = 0.018), which is calculated to lie lower in energy than the S_0_→S_1_ transition of (Cyc-DMBI)_2_ and presumably corresponds to the shoulder observed in the experimental spectrum of (N-DMBI)_2_, and S_0_→S_5_, which lies very close to S_0_→S_6_. For both of these transitions, the NTOs indicate considerable charge transfer from the bibenzoimidazole portion of the molecules to the C_6_H_4_NMe_2_ Y substituents (see Figure 3d and Supporting Information File 1, Figure S11).

**Figure 4:**
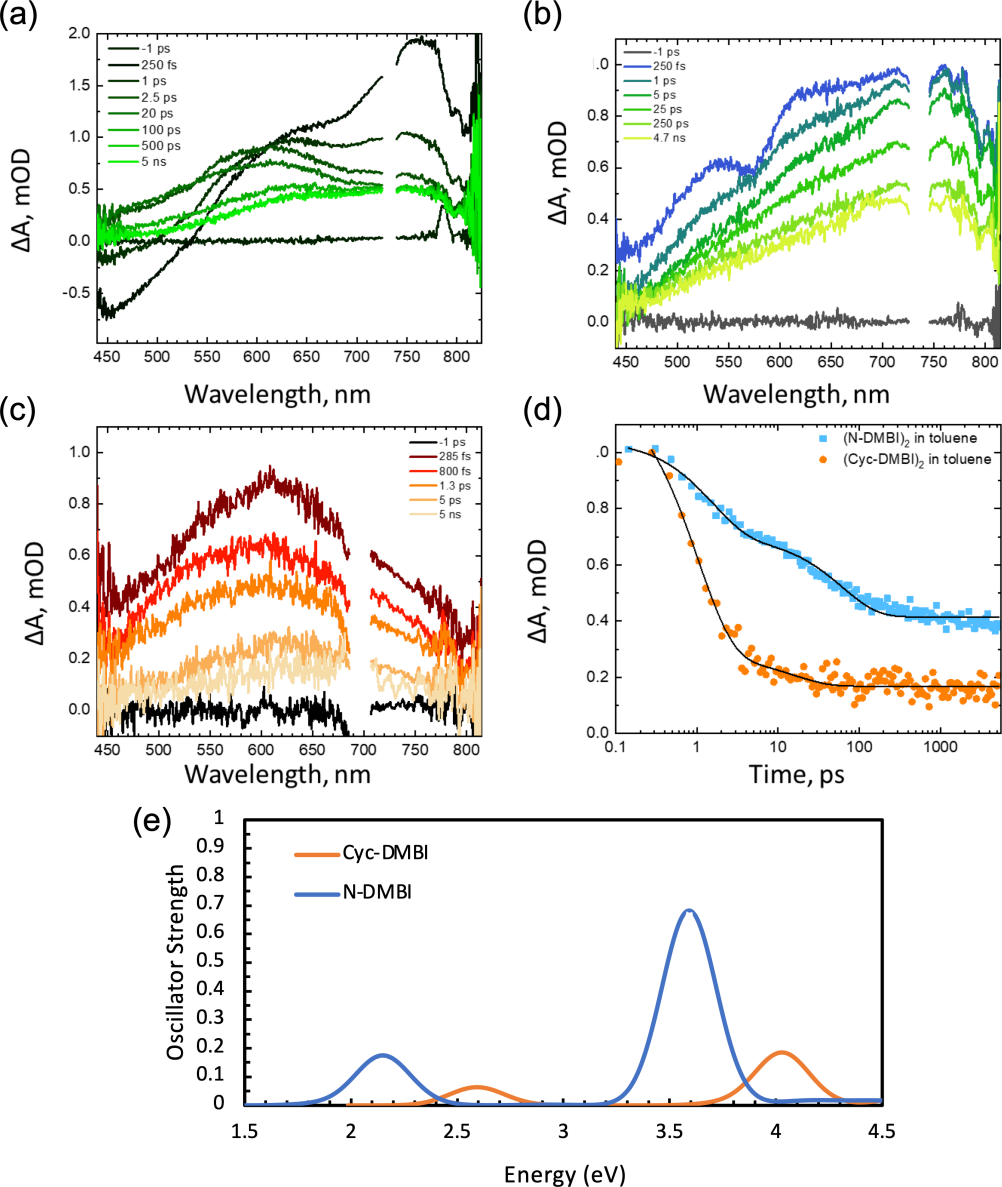
Transient absorption spectra of (a) (N-DMBI)_2_ in MeCN, (b) (N-DMBI)_2_ in toluene, and (c) (Cyc-DMBI)_2_ in toluene with excitation at 350 nm in the absence of oxygen. (d) Comparison of temporal evolution of the absorption at 654 nm for both dimers in toluene. (e) Spectra for the two monomeric radicals obtained from M06/6-31G(d,p) TD-DFT calculations.

Most of the above-mentioned substrates exhibit little or no absorption at 365 nm (see Supporting Information File 1, Figure S4 for comparison of spectra of the reductants and BnBr); thus, although slight overlap between the tail of substrate absorption and the tail of the output of the nominally 365 nm LED may be responsible for the reactivity seen in the absence of dimeric reductants, the stronger absorption of the two dimeric reductants examined suggests photo-acceleration of the reactions involves the excited states of the dimers. In principle excitation of the dimers might result in cleavage of the dimers to the corresponding radical monomers, D^•^, which can then reduce RX, or to ET from dimer excited states, D_2_*, to RX. The results in Table 2 support the former effect: if photoinduced cleavage occurs we would expect the scope of RX cleavage to be more-or-less limited by the reducing strength of D^•^ (*E*(D^+^/D^•^) = ca. −2.4 V for both species used here), whereas the singlet excited state of the dimer, D_2_*, should be more reducing, allowing more challenging substrates to be reduced (*E*(D_2_^•+^/D_2_*) can be estimated as ca. −3.4 and −2.8 V for (Cyc-DMBI)_2_ and (Cyc-DMBI)_2_, respectively, using values of *E*(D_2_^•+^/D_2_) and estimated absorption onsets). Furthermore, the NTOs for the strong absorptions of both dimers and the weak low-energy absorption of (N-DMBI)_2_ both involve some depopulations of the bibenzoimidazole-based HOMO, which, as well as π-character also has significant C–σ-bonding character associated with the inter-monomer bond, suggesting that excitation should weaken the bond (in a similar way to oxidation associated with removal of an electron from the same orbital). To investigate this possibility in more detail we recorded transient absorption spectra of the dimers following excitation at 350 nm. Importantly, the samples did not degrade during the experiment, indicating that the species observed eventually reform the dimers. The transient absorption spectra of (N-DMBI)_2_ (Figure 4a) show significant spectral evolution within the first 1 ps following photoexcitation at 350 nm in MeCN. The (negative) bleach feature at 450 nm and photoinduced absorption (PIA) peaking at 775 nm transform into a broad PIA spanning the visible wavelengths with a notable isosbestic point at 600 nm. There is a secondary evolution occurring with a time constant of approximately 60 ps resulting in a long-lived feature existing beyond the 5 ns window of the ultrafast experiment. In toluene (Figure 4b), the spectral features at early times are different; however, the kinetics at 654 nm are the same between solvents suggesting that the initial features dominating the spectra at 250 fs in MeCN are present in toluene.

The (Cyc-DMBI)_2_ exhibits faster decay kinetics (Figure 4c) than (N-DMBI)_2_ under the same experimental conditions. The spectral shape is similar to the late time spectra of the (N-DMBI)_2_ with a broad PIA spanning the visible wavelengths, which may suggest that the initial evolution observed in the (N-DMBI)_2_ occurs within our instrument response. In toluene, the signal amplitude at 5 ns for (Cyc-DMBI)_2_ is significantly smaller than that of the (N-DMBI)_2_ and 96% of the signal amplitude at 654 nm decays with a 1 ps lifetime. The isosbestic point in Figure 4a is consistent with a clean transformation such as monomer formation. The corresponding monomeric radicals are calculated using TD-DFT (M06/6-31G(d,p)) (Figure 4e) to have absorptions at similar energies to those seen in the longer-time spectra with Cyc-DMBI^•^ having a blue-shifted absorption relative to N-DMBI^•^, consistent with the observed features arising from the monomeric radicals. Intersystem crossing (ISC) from the singlet to triplet excited state is also a possibility and TD-DFT calculations suggest that the dimer T_1_ states absorb at similar wavelengths to the monomer radicals (see Supporting Information File 1, Figure S12). However, ISC is typically much slower than 1 ps in organic molecules, especially so for those lacking heavy atoms [45].

## Conclusion

Dimeric reductants of the (Y-DMBI)_2_ type can accomplish the dehalogenation of a variety of organic halides with peak reduction potentials less cathodic than ca. −2.0 V vs FeCp_2_^+/0^, affording essentially quantitative conversions in a few hours under 365 nm illumination. In the case of benzyl halides the primary products are bibenzyls, some of which may be challenging to synthesize by other methods, whereas aryl halides afford dehalogenated arenes. One example also demonstrates that the approach may have utility for selectively dehalogenating compounds containing different halogens. The photoacceleration of these reactions appears to occur through photocleavage of the dimers to the more strongly reducing monomers, which may help inform the use of these and related reductants in achieving other chemical transformations.

## Supporting Information

File 1 Synthetic and other experimental procedures, additional data, additional TD-DFT results, and NMR spectra of compounds.

## References

[1] Murphy J A, Khan T A, Zhou S-z, Thomson D W, Mahesh M (2005). Angew Chem, Int Ed.

[2] Murphy J A, Zhou S-z, Thomson D W, Schoenebeck F, Mahesh M, Park S R, Tuttle T, Berlouis L E A (2007). Angew Chem, Int Ed.

[3] Murphy J A, Garnier J, Park S R, Schoenebeck F, Zhou S-z, Turner A T (2008). Org Lett.

[4] Hanson S S, Doni E, Traboulsee K T, Coulthard G, Murphy J A, Dyker C A (2015). Angew Chem, Int Ed.

[5] Zhou S, Anderson G M, Mondal B, Doni E, Ironmonger V, Kranz M, Tuttle T, Murphy J A (2014). Chem Sci.

[6] Huang M, Tang J, Kim J K, Gong M, Zhang J, Li Y, Wu Y (2022). Org Biomol Chem.

[7] Herberich G E, Bauer E, Schwarzer J (1969). J Organomet Chem.

[8] Narayanam J M R, Tucker J W, Stephenson C R J (2009). J Am Chem Soc.

[9] Furst L, Matsuura B S, Narayanam J M R, Tucker J W, Stephenson C R J (2010). Org Lett.

[10] Ghosh I, Ghosh T, Bardagi J I, König B (2014). Science.

[11] Constantin T, Zanini M, Regni A, Sheikh N S, Juliá F, Leonori D (2020). Science.

[12] Tintori G, Fall A, Assani N, Zhao Y, Bergé-Lefranc D, Redon S, Vanelle P, Broggi J (2021). Org Chem Front.

[13] Zhou S, Doni E, Anderson G M, Kane R G, MacDougall S W, Ironmonger V M, Tuttle T, Murphy J A (2014). J Am Chem Soc.

[14] Ludvík J, Volke J, Pragst F (1986). J Electroanal Chem Interfacial Electrochem.

[15] Ludvík J, Pragst F, Volke J (1984). J Electroanal Chem Interfacial Electrochem.

[16] Pragst F, Niazymbetov M (1986). J Electroanal Chem Interfacial Electrochem.

[17] Naab B D, Zhang S, Vandewal K, Salleo A, Barlow S, Marder S R, Bao Z (2014). Adv Mater (Weinheim, Ger).

[18] Zhang S, Naab B D, Jucov E V, Parkin S, Evans E G B, Millhauser G L, Timofeeva T V, Risko C, Brédas J-L, Bao Z (2015). Chem – Eur J.

[19] Naab B D, Gu X, Kurosawa T, To J W F, Salleo A, Bao Z (2016). Adv Electron Mater.

[20] Yuan D, Huang D, Zhang C, Zou Y, Di C-a, Zhu X, Zhu D (2017). ACS Appl Mater Interfaces.

[21] Schwarze M, Gaul C, Scholz R, Bussolotti F, Hofacker A, Schellhammer K S, Nell B, Naab B D, Bao Z, Spoltore D (2019). Nat Mater.

[22] Al Kurdi K, Gregory S A, Jhulki S, Conte M, Barlow S, Yee S K, Marder S R (2020). Mater Adv.

[23] Un H-I, Gregory S A, Mohapatra S K, Xiong M, Longhi E, Lu Y, Rigin S, Jhulki S, Yang C-Y, Timofeeva T V (2019). Adv Energy Mater.

[24] Lungwitz D, Joy S, Mansour A E, Opitz A, Karunasena C, Li H, Panjwani N A, Moudgil K, Tang K, Behrends J (2023). J Phys Chem Lett.

[25] Pham P H, Barlow S, Marder S R, Luca O R (2023). Chem Catal.

[26] Mohapatra S K, Marder S R, Barlow S (2022). Acc Chem Res.

[27] (2023). https://www.hepatochem.com/product/hck1012-xx-011/.

[28] Newcomb M, Park S U (1986). J Am Chem Soc.

[29] Bietti M, Martella R, Salamone M (2011). Org Lett.

[30] Jing L, Nash J J, Kenttämaa H I (2008). J Am Chem Soc.

[31] Higginson B, Sanjosé-Orduna J, Gu Y, Martin R (2021). Synlett.

[32] Yu D, To W-P, Tong G S M, Wu L-L, Chan K-T, Du L, Phillips D L, Liu Y, Che C-M (2020). Chem Sci.

[33] Inaba S, Matsumoto H, Rieke R D (1984). J Org Chem.

[34] Burns T P, Rieke R D (1987). J Org Chem.

[35] Kim S-H, Rieke R D (2000). J Org Chem.

[36] Suh Y, Lee J-s, Kim S-H, Rieke R D (2003). J Organomet Chem.

[37] Wiesner S, Walter P, Wagner A, Kaifer E, Himmel H-J (2016). Eur J Org Chem.

[38] Blanksby S J, Ellison G B (2003). Acc Chem Res.

[39] Mohapatra S K, Al Kurdi K, Jhulki S, Bogdanov G, Bacsa J, Conte M, Timofeeva T V, Marder S R, Barlow S (2023). Beilstein J Org Chem.

[40] Guo S, Mohapatra S K, Romanov A, Timofeeva T V, Hardcastle K I, Yesudas K, Risko C, Brédas J-L, Marder S R, Barlow S (2012). Chem – Eur J.

[41] Longhi E, Risko C, Bacsa J, Khrustalev V, Rigin S, Moudgil K, Timofeeva T V, Marder S R, Barlow S (2021). Dalton Trans.

[42] Bennett R W, Wharry D L, Koch T H (1980). J Am Chem Soc.

[43] Colter A K, Lai C C, Parsons A G, Ramsey N B, Saito G (1985). Can J Chem.

[44] Jhulki S, Un H-I, Ding Y-F, Risko C, Mohapatra S K, Pei J, Barlow S, Marder S R (2021). Chem.

[45] Imran M, Zhang X, Wang Z, Chen X, Zhao J, Barbon A, Voronkova V K (2021). Phys Chem Chem Phys.

