# Supplementary material for: Beyond n-dopants for organic semiconductors: use of bibenzo[d]imidazoles in UV-promoted dehalogenation reactions of organic halides
Source: Beilstein J Org Chem. 2023 Dec 14;19:1912–22. doi: 10.3762/bjoc.19.142 (PMC10729154; doi:10.3762/bjoc.19.142)
Supplement: File 1 — Synthetic and other experimental procedures, additional data, additional TD-DFT results, and NMR spectra of compounds. [file Beilstein_J_Org_Chem-19-1912-s001.pdf]

BEILSTEIN JOURNAL OF ORGANIC CHEMISTRY

Supporting Information

for

Beyond n-dopants for organic semiconductors: use of bibenzo[*d*]imidazoles in UV-promoted dehalogenation reactions of organic halides

Kan Tang, Megan R. Brown, Chad Risko, Melissa K. Gish, Garry Rumbles, Phuc H. Pham, Oana R. Luca, Stephen Barlow and Seth R. Marder

Beilstein J. Org. Chem. **2023,** *19,* 1912–1922. doi:10.3762/bjoc.19.142

Synthetic and other experimental procedures, additional data, additional TD-DFT results, and NMR spectra of compounds

License and Terms: This is a supporting information file under the terms of the Creative Commons Attribution License (https://creativecommons.org/licenses/by/4.0). Please note that the reuse, redistribution and reproduction in particular requires that the author(s) and source are credited and that individual graphics may be subject to special legal provisions.
The license is subject to the *Beilstein Journal of Organic Chemistry* terms and conditions: (https://www.beilstein-journals.org/bjoc/terms)

Table of contents

1. General experimental details S2

2. Synthesis of 2,2’-disubtituted-1,1',3,3'-tetramethyl-2,2',3,3'-tetrahydro-2,2'-bibenzo[*d*]imidazoles, $(Y\text{-}DMBI)_2$, and related compounds S2

3. Reaction set-up, characterization, and quantification S4

   3.1. General procedure for the dehalogenation reactions and quantification S4

   3.2. Additional results on the scope of the dehalogenation reactions S6

   3.3. Procedure for the kinetic study of benzyl bromide dehalogenation in the dark S11

   3.4. Results on the kinetic study of benzyl bromide dehalogenation in the dark S13

   3.5. Synthesis and isolation of dimeric coupling product for GC quantification S14

4. UV–vis spectra of benzyl bromide and dimers S15

5. Cyclic voltammograms of organic halides S18

6. NMR crossover experiments S20

7. TD-DFT calculations S22

8. Transient absorption spectroscopy S24

9. NMR Spectra S25

1. General experimental details

All commercially available fine chemicals were used without further purification unless otherwise noted. All operations involved in the synthesis of the dimers were performed under a nitrogen atmosphere using standard Schlenk techniques. Anhydrous THF was obtained from a solvent purification system (MBraun). Anhydrous toluene and hexane used in the dimer synthesis were purchased from Sigma Aldrich. Sodium amalgam (1 wt % Na:Hg) was prepared prior to the reactions by the addition of small pieces of Na metal to vigorously stirred Hg (Sigma, Aldrich, electronic grade, 99.99%). The dehalogenation reaction mixtures were prepared in a nitrogen-filled glovebox (Vac. Atm. Co.). The reactions were carried out in a HepatoChem photoreactor (EvoluChem PhotoRedOx Box TC™ photoreactor using a 365 nm 9 mW cm^{-2} LED light source. ^{1}H and ^{13}C{^{1}H} NMR spectra were recorded on a Bruker Avance-III 400 NMR or a Varian INOVA 400 NMR spectrometer and were referenced to tetramethylsilane (TMS) using the residual proton or carbon signal of the deuterated solvent, respectively. The solutions for vis/NIR measurements of the dimers were prepared by dissolving dimers in THF inside a glove box. The solutions (10^{-6} to 10^{-3} M) were transferred into PTFE stopcock-sealed quartz cuvettes with path lengths of 1 cm. The cuvettes were then sealed and taken out of the glove box for the measurements by an Agilent Cary 5000 spectrometer. Cyclic voltammetry was performed in 0.1 M *n*-Bu_4NPF_6 in anhydrous THF under a steady flow of nitrogen over the solution using a CH Instruments 620D potentiostat. A three-electrode configuration was adopted where a glassy carbon disk working electrode (dia. 3 mm), a platinum wire counter electrode, and an Ag/Ag^+ reference electrode (Ag wire submerged in 10 mM $AgNO_3$/0.1 M *n*Bu_4NPF_6 acetonitrile solution) were used. A scan rate of 100 mV s^{-1} was used and ferrocene was used as an internal reference.

2. Synthesis of 2,2'-disubtituted-1,1',3,3'-tetramethyl-2,2',3,3'-tetrahydro-2,2'-bibenzo[*d*]imidazoles, (Y-DMBI)$_2$, and related compounds

4-(1*H*-Benzimidazol-2-yl)-*N,N*-dimethylaniline: A mixture of the 1,2-phenylenediamine (5 g, 46.3 mmol), 4-(dimethylamino)benzaldehyde (6.9 g, 46.3 mmol), and sodium metabisulfite (55.56 mmol, 1.2 equiv, 10.5 g) was added to a flask. The flask was flushed with N_2, after which dry DMF (100 mL) was added. The mixture was heated to 100 °C and stirred under N_2. After overnight reaction (ca. 16 h), water (ca. 100 mL) was added to the reaction mixture, leading to the formation of a light yellow precipitate. The solid was collected by filtration, then re-dispersed in

EtOAc and heated at 70 °C with stirring for 1 h to remove any unreacted starting materials. After cooling, the solid was collected by vacuum filtration and dried under vacuum overnight to afford a light-yellow solid (9.6 g, 87%). ^{1}H NMR (400 MHz, DMSO-d_6): δ 8.01 (dd, J = 8.9, 1.7 Hz, 2H), 7.59–7.48 (m, 2H), 7.26–7.11 (m, 2H), 6.86 (dd, J = 8.9, 1.7 Hz, 2H), 3.02 (s, 6H).

N-DMBI$^+$PF$_6^-$: 4-(1*H*-Benzimidazol-2-yl)-*N,N*-dimethylaniline (4.54 g, 19.1 mmol), iodomethane (8.54 g, 60 mmol, 3.7 mL), and K_2CO_3 (14 g, 57.4 mmol) were added to acetone (190 mL) in a 250 mL Chemglass pressure flask, which was then sealed and heated to 100 °C with stirring for 24 h, over which time the slurry turned from yellow to white. After allowing to cool, the acetone was removed using rotary evaporation and the white powder (N-DMBI$^+$I$^-$) was washed with water and hexane and then dissolved in a mixture of EtOH/water 5:1 (450 mL) with vigorous stirring at 60 °C. NH_4PF_6 (5.5 g, 34 mmol) was added to the clear solution, and a white precipitate formed immediately. After stirring for ca. 1 h, the solid was collected by vacuum filtration and dried under vacuum overnight to afford a white powder (6.5 g, 83%). ^{1}H NMR (400 MHz, acetone-d_6): δ 8.10–7.96 (m, 2H), 7.87–7.69 (m, 4H), 7.16–6.99 (m, 2H), 4.11 (s, 6H), 3.26 (s, 6H).

(N-DMBI)$_2$: The synthesis of the (N-DMBI)$_2$ from the reduction of N-DMBI$^+$PF$_6^-$ by 1 wt % Na:Hg was performed by following the reported procedures [[1]]. A slurry of N-DMBI$^+$PF$_6^-$ (3.1 g, 8.8 mmol) was stirred in THF with 1 wt % Na:Hg (from 870 mg Na in 87 g Hg) for 2.5 h at room temperature under nitrogen. An off-white solid (1.7 g, yield 85%) was obtained. ^{1}H NMR (400 MHz, benzene-d_6): δ 7.42 (br, 4H), 6.97–6.76 (br, 4H), 6.47–6.01 (br, 8H), 2.72–2.30 (m, br, 24H).

4-(1*H*-Benzimidazol-2-yl)-cyclohexane: The synthesis of the 4-(1*H*-benzimidazol-2-yl)-cyclohexane is similar to the aforementioned synthetic protocol of 4-(1*H*-benzimidazol-2-yl)-*N*,*N*-dimethylaniline. A mixture of the 1,2-phenylenediamine (5 g, 46.3 mmol), cyclohexanecarboxaldehyde (5.2 g, 46.3 mmol, 5.6 mL), and sodium metabisulfite (55.56 mmol, 1.2 equiv, 10.5 g) was added to a flask and stirred at 100 °C under N_2 overnight. A white solid product (4.6 g, 50%) was obtained. ^{1}H NMR (400 MHz, DMSO-d_6): δ 12.09 (s, 1H), 7.64–7.30 (m, br, 2H), 7.30–6.96 (m, 2H), 2.99–2.73 (m, 1H), 2.19–1.13 (m, br, 10H).

2-Cyc-DMBI$^+$PF$_6^-$: The synthesis of the 2-cyc-DMBI$^+$PF$_6^-$ is similar to the synthetic protocol of N-DMBI$^+$PF$_6^-$. 4-(1*H*-Benzimidazol-2-yl)-cyclohexane (3.0 g, 15 mmol), iodomethane (6.57 g, 46 mmol, 2.8 ml), and K_2CO_3 (11 g, 44.2 mmol) in 190 mL of acetone was stirred at 100 °C for

24 h. A white powder (5.2 g, 95%) was obtained after drying under vacuum overnight. ^{1}H NMR (400 MHz, DMSO-d_6): δ 8.09–7.97 (m, 2H), 7.72–7.61 (m, 2H), 4.12 (s, 6H), 3.56–3.43 (m, 1H), 2.08–1.38 (m, 10H).

(2-Cyc-DMBI)$_2$: The synthesis of the (2-cyc-DMBI)$_2$ from the reduction of 2-cyc-DMBI^{+}PF$_6^{-}$ by 1 wt % Na:Hg was performed by following the reported procedures [2]. A slurry of Cyc-DMBI^{+}PF$_6^{-}$ (2.0 g, 5.3 mmol) was stirred in THF with 1 wt % Na:Hg (from 600 mg Na in 60 g Hg) for 2.5 h at room temperature under nitrogen. A white solid (0.78 g, 65%) was obtained. ^{1}H NMR (400 MHz, benzene-d_6): δ 6.73 (dd, J = 5.3, 3.1 Hz, 4H), 6.06 (dd, J = 5.3, 3.2 Hz, 4H), 1.75–1.66 (m, 2H), 1.47 (d, J = 6.8 Hz, 10H), 0.94 (s, 10H).

3. Reaction set-up, characterization, and quantification

3.1. General procedure for the dehalogenation reactions and quantification

In a nitrogen-filled glovebox, a 215 mM stock solution of the organic halide in anhydrous THF was prepared. Liquid halides were subjected to three freeze-pump-thaw cycles in a Schlenk tube before being transferred into the glovebox. Typically, a PTFE-lined glass vial with a stirring bar was charged with 20 mg (0.0375 mmol) of the appropriate (Y-DMBI)$_2$ dimer and 3.65 mL of anhydrous (stabilizer-free) THF. Then, 0.075 mmol of the halide substrate was added by pipetting 348 μL of the stock solution into the vial resulting in a 4 mL solution (RX, 18.7 mM). The vial was taken out of the glovebox immediately after the mixing and subjected to various reaction conditions (i.e., light, time). The reactions were carried out in a HepatoChem photoreactor (EvoluChem PhotoRedOx Box TC™ photoreactor fitted with a 365 nm 9 mW cm^{-2} LED light source at room temperature. The reaction setup is shown in Figure S1, the reaction vials were immersed in a water bath at room temperature to minimize the temperature elevation. The temperature increase after 4 h of UV irradiation is ca. 5 °C (from ca. 20 to 25 °C).

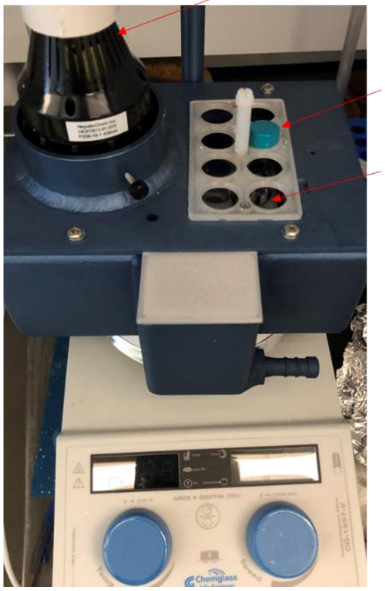

Figure S1. The photoreactor setup used in the dehalogenation reactions.

After the reaction was completed, an aliquot was taken of the reaction mixture (0.1 to 0.2 mL) using a syringe and passed through a 0.25 μm PTFE filter into a 2 ml GC–MS vial that was preloaded with 1–2 μL of decane as internal standard. The clear aliquot was then diluted at least 10 times by THF (ACS grade, stabilized with BHT). The resulting sample was introduced to the GC–MS. The reactant peak and product peaks were characterized and quantified by the calibration curves established from the extracted ion chromatogram (EIC) of each substrate and product. Reactions of 4-methylbenzyl chloride were quantified by GC–FID.

The conversion of the reaction was calculated as

$$Conversion = ([RX]_0 - [RX])/[RX]_0 * 100\%$$

The yield of the H-abstraction product (RH) was calculated as,

$$yield\ (RH) = [RH]/[RX]_0 * 100\%$$

The yield of the dimeric coupling product (R_2) was calculated as,

$$yield\ (R2)\ = 2[RR]/[RX]_0 * 100\%$$

3.2. Additional results on the scope of the dehalogenation reactions

Table S1. Debromination of benzyl and alkyl halides.

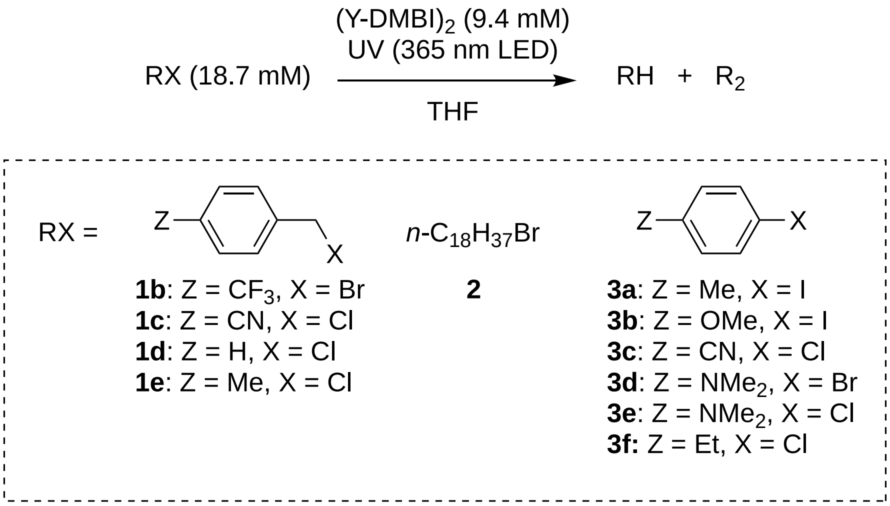

Entry	RX, E_{pc} / V[a]	Rn. Time / h	UV	Reductant	Conversion / %	RH yield / %	R_2 yield / %
1	**1b**, −1.5	2	No	$(N\text{-}DMBI)_2$	22	5	16
2		2	Yes	–	8	7	1
3		2	Yes	$(N\text{-}DMBI)_2$	77	2	71
4		4	Yes	$(N\text{-}DMBI)_2$	80	2	75
5		6	Yes	$(N\text{-}DMBI)_2$	100	trace	>98
6	**1c**, −1.6	2	No	$(N\text{-}DMBI)_2$	52	34	17
7		2	Yes	–	2	b	b
8		2	Yes	$(N\text{-}DMBI)_2$	100	13	87
9		4	Yes	$(N\text{-}DMBI)_2$	100	14	86
10	**1d**, −2.3	2	No	$(N\text{-}DMBI)_2$	5	4	1
11		2	Yes	–	<1	<1	0
12		2	Yes	$(N\text{-}DMBI)_2$	35	7	4

13		4	Yes	$(N\text{-}DMBI)_2$	39	7	5
14	**1e**[c], −2.4	2	No	$(N\text{-}DMBI)_2$	31	2	4
15		2	Yes	–	4	b	b
16		2	Yes	$(N\text{-}DMBI)_2$	66	2	3
17		4	Yes	$(N\text{-}DMBI)_2$	73	3	3
18		2	No	$(Cyc\text{-}DMBI)_2$	7.5	3	1.5
19		2	Yes	$(Cyc\text{-}DMBI)_2$	52	3	27
20		4	Yes	$(Cyc\text{-}DMBI)_2$	60	3	32
21		20	Yes	$(Cyc\text{-}DMBI)_2$	65	3	35
22	**2**, −2.1	2	No	$(N\text{-}DMBI)_2$	0	0	d
23		2	Yes	–	10	5	d
24		2	Yes	$(N\text{-}DMBI)_2$	18	9	d
25		4	Yes	$(N\text{-}DMBI)_2$	39	19	d
26	**3a**, −1.8	1	No	$(N\text{-}DMBI)_2$	41	41	b
27		1	Yes	$(N\text{-}DMBI)_2$	93	93	b
28		2	No	$(N\text{-}DMBI)_2$	40	41	b
29		2	Yes	–	6	6	b
30		2	Yes	$(N\text{-}DMBI)_2$	98	98	b
31	**3b**, −2.3	1	No	$(N\text{-}DMBI)_2$	30	30	b
32		1	Yes	$(N\text{-}DMBI)_2$	94	94	b
33		2	No	$(N\text{-}DMBI)_2$	39	39	b
34		2	Yes	–	5.5	5.5	b
35		2	Yes	$(N\text{-}DMBI)_2$	99	99	b
36	**3c**, −1.6	2	No	$(N\text{-}DMBI)_2$	31	7.5	b
37		2	Yes	–	2.4	0	b

38		2	Yes	$(N\text{-}DMBI)_2$	57	41	b
39		4	Yes	$(N\text{-}DMBI)_2$	55	41	b
40		2	No	$(Cyc\text{-}DMBI)_2$	28	0	b
41		2	Yes	$(Cyc\text{-}DMBI)_2$	94	59	b
42		4	Yes	$(Cyc\text{-}DMBI)_2$	99	60[e]	b
43	**3d**, −2.3	2	No	$(N\text{-}DMBI)_2$	6	6	b
44		2	Yes	–	2	0	b
45		2	Yes	$(N\text{-}DMBI)_2$	5	5	b
46		4	Yes	$(N\text{-}DMBI)_2$	5	5	b
47		2	None	$(Cyc\text{-}DMBI)_2$	1	0	b
48		2	Yes	$(Cyc\text{-}DMBI)_2$	1	1	b
49		4	Yes	$(Cyc\text{-}DMBI)_2$	5	5	b
50	**3e**, −2.3	2	No	$(N\text{-}DMBI)_2$	1	0	b
51		2	Yes	–	3	0	b
52		2	Yes	$(N\text{-}DMBI)_2$	4	0	b
53		4	Yes	$(N\text{-}DMBI)_2$	4	0	b
54		2	No	$(Cyc\text{-}DMBI)_2$	2	0	b
55		2	Yes	$(Cyc\text{-}DMBI)_2$	0	0	b
56		4	Yes	$(Cyc\text{-}DMBI)_2$	2	2	b
57	**3f**, −2.3	2	No	$(N\text{-}DMBI)_2$	0	0	b
58		2	Yes	–	0	0	b
59		2	Yes	$(N\text{-}DMBI)_2$	0	0	b
60		4	Yes	$(N\text{-}DMBI)_2$	1	1	b
61		2	No	$(Cyc\text{-}DMBI)_2$	0	0	b

62	2	Yes	$(Cyc\text{-}DMBI)_2$	1	0	b
63	4	Yes	$(Cyc\text{-}DMBI)_2$	<1	<1	b

[a]Peak reduction potential *vs*. $FeCp_2^{+/0}$ in THF / 0.1 M Bu_4NPF_6 (see Figure S7 and S8 for cyclic voltammograms). [b]Not detected. [c]Quantified by GC–FID; see also Table S2 and Figure S2. [d]Likely not detectable by GC–MS. [e]65% after 20 h.

Table S2. Dehalogenation of 4-methylbenzyl chloride (**1e**) using anhydrous toluene as solvent. Conditions: [4-methylbenzyl chloride] = 18.7 mM, UV illumination (365 nm) as indicated.[a]

Entry	Reaction time (h)	UV illumination	$[(2\text{-}Cyc\text{-}DMBI)_2]$ (mM)	Conversion (%)	RH Yield (%)	R_2 Yield (%)
1	2	No	9.4	2	0	<1
2	2	Yes	9.4	73	0	61
3	4	Yes	9.4	96	0	65
4	20	Yes	9.4	100	0	70

[a]See Figure S2.

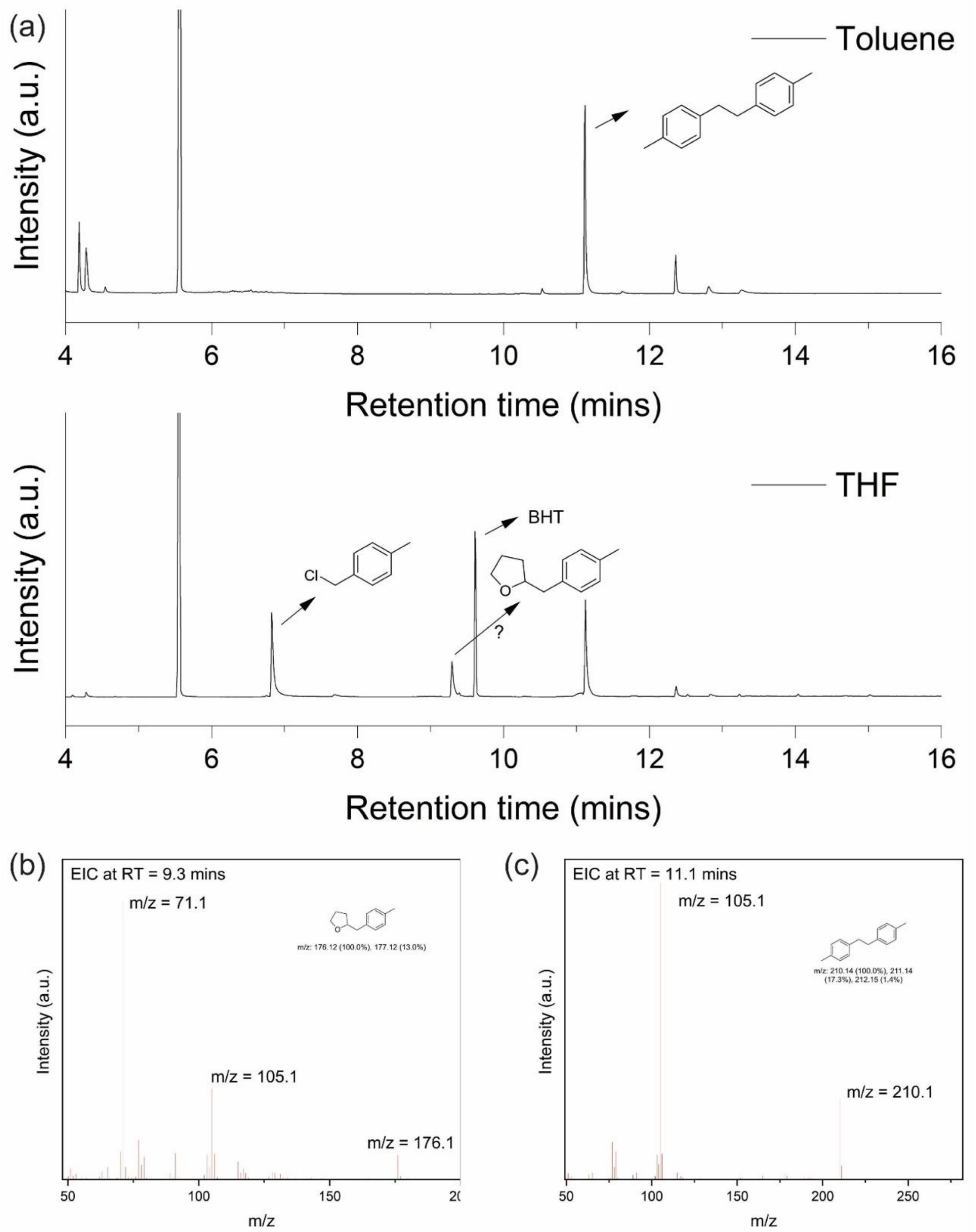

Figure S2. (a) GC–MS chromatograms of the dehalogenation reaction mixture of 4-methylbenzyl chloride in anhydrous toluene (top, entry 3 in Table S2) and anhydrous THF (bottom, entry 20 in Table S1). In the case of THF, aside from the major coupling product at retention time (RT) = 11.1 min, there is a peak at 9.3 min not seen in toluene, which is assigned to the 4-methylbenzyl–THF adduct (or an isomer of this compound) after analyzing the fragmentation patterns in panel (b) and (c). Note that the peaks at RT= 5.7 min are the decane internal standard and that the BHT originates in the stabilized THF used to dilute the reaction mixture prior to injection into the GC–MS rather than the unstabilized solvent in which the reaction was run. The position of substitution on THF was not experimentally established.

Table S3. Dehalogenation of benzyl bromide (BnBr) in the presence of equimolar TMSCl. Conditions: [BnBr] = [TMSCl] = 18.7 mM, anhydrous THF as the solvent, UV illumination (365 nm) as indicated.[a]

Reaction time (h)	UV illumination	$[(N\text{-}DMBI)_2]$ (mM)	Conversion (%)	BnH Yield (%)	Bn_2 Yield (%)	BnCl Yield
4	Yes	9.4	46	6	30	10

[a]No Me_3SiBn detected suggesting that Bn^- is not formed. BnCl is presumably formed by Cl abstraction from TMSCl by $Bn^{\bullet}$.

Table S4. Dehalogenation of bifunctional 2-iodobenzyl chloride. Conditions: 2-iodobenzyl chloride is 18.7 mM, anhydrous THF as solvent.

Reaction time (h)	UV illumination	$[N\text{-}DMBI_2]$ (mM)	Conversion (%)	BnCl Yield (%)	2-Iodotoluene Yield (%)
2	No	9.4	17	17	0
2	Yes	9.4	97	97	0
4	Yes	9.4	98	98	0
2	Yes	0	5	5	0

3.3. Procedure for the kinetic study of benzyl bromide dehalogenation in the dark

In a nitrogen-filled glovebox, a 215 mM benzyl bromide stock solution in anhydrous THF was prepared before the reactions. The dimeric reductant and benzyl bromide stock solution were added sequentially into a 50 mL Schlenk flask with a stirring bar to reach the desired amount of each component in the different groups of the kinetic study. The reaction mixture was diluted to 20 mL using anhydrous THF. The Schlenk flask was taken out of the glovebox immediately after the mixing and connected to an N_2 line after pump–filling the hose three times. 0.1 to 0.2 mL aliquots of the reaction mixture were withdrawn during the reaction and filtered into a GC–MS vial. The quantification steps of the sample are identical to the ones in the aforementioned single dehalogenation reaction. The conversion and yield calculations are identical to those in the above-mentioned reactions. The electron-transfer efficiency (ETF) was calculated as,

$$ETF = \frac{2[RR] + [RH]}{[[RX]_0, 2[D_2]]_{min}} * 100\%$$

The denominator is defined as the amount of either the halide substrate or two times the dimer, depending on which one is smaller as a limiting factor in the reductive dehalogenation. An ideal 100% ETF indicates that all electrons transferred from the dimer were utilized in the reduction of the substrate.

Table S5. Summary of the conversions and yields of the dehalogenation reaction of benzyl bromide. Conditions: anhydrous THF as solvent, dark.

Entry	Reaction time (h)	[BnBr] (mM)	[(Y-DMBI)$_2$] (mM)	Conversion (%)	Toluene yield (%)	Bibenzyl yield (%)	Electron transfer efficiency (%)
			(N-DMBI)$_2$				
1	50	7.35	1.5	36	6	29	88
2	55	5.25	1.5	55	9	49	96
3	96	3	1.5	95	16	77	95
4	30	1.5	1.5	98	22	78	98
			(2-Cyc-DMBI)$_2$				
5	28	7.35	1.5	14	5	9.5	34
6	46	5.25	3.75	19	9	10	19
7	22	3	1.5	15	8	6.4	15
8	26	1.5	1.5	14	10	4	14

3.4. Results on the kinetic study of benzyl bromide dehalogenation in the dark

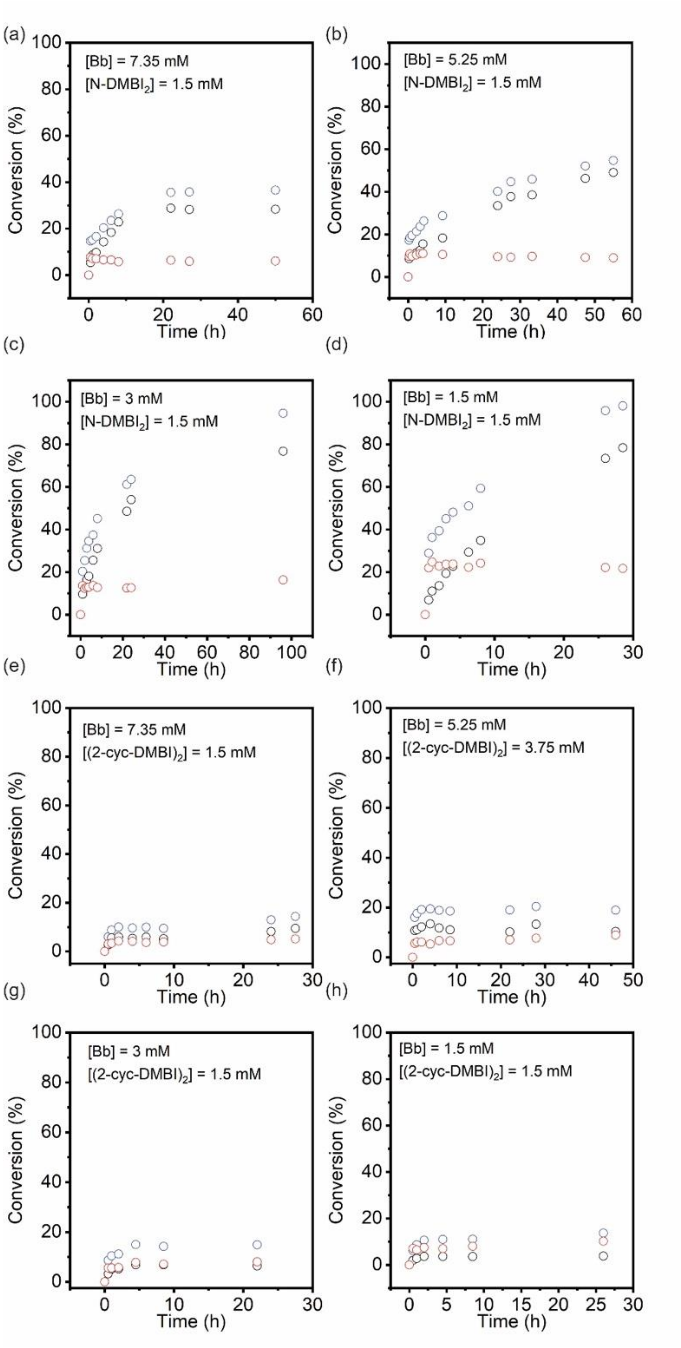

Figure S3. Kinetic studies on the conversions and yields of the dehalogenation reaction of benzyl bromide (Bb). (a) 7.35 mM benzyl bromide. (b) 5.25 mM benzyl bromide. (c) 3 mM benzyl bromide. (d) 1.5 mM benzyl bromide. Blue dots: conversions, black dots: bibenzyl yield, red dots: toluene yield. Conditions: anhydrous THF as the solvent, dark. The reactions were conducted in a Schlenk flask, and the aliquots were taken during the proceeding of the reaction for GC–MS analysis. The conversions and yields are based on the amount of benzyl bromide substrate.

3.5. Synthesis and isolation of dimeric coupling product for GC quantification

All of the H-abstraction products and some of the bibenzyl derivatives are commercially available and were directly used for GC–MS quantification. 4,4'-(1,2-Ethanediyl)dibenzonitrile and 1,1'-(1,2-ethanediyl)bis[4-(trifluoromethyl)benzene] were synthesized as described below and isolated separately for GC–MS calibration. In both cases, the compounds have been reported in the literature many times and the NMR data are consistent with those previously reported, for example in ref. [3], where they were made from the corresponding benzyl bromides using a tungsten containing photocatalyst.

4,4'-(1,2-Ethanediyl)dibenzonitrile: A 50 mL Schlenk flask with a stirring bar was charged with naphthalene (0.42 g, 3.3 mmol) and anhydrous THF (20 mL). Under a flow of N_2, freshly cut sodium metal (ca. 80 mg, 3.5 mmol) was added to the naphthalene solution, and the solution was ultrasonicated for 30 min. After stirring for 2 h, the THF solution turned completely dark; it was then transferred to a THF solution containing 4-(chloromethyl)benzonitrile (0.50 g, 3.3 mmol) via a cannula. The black solution was stirred overnight for 20 h. After quenching the reaction with the addition of excess MeOH, the solvent was removed by rotavap without heating. Dichloromethane (ca. 2 mL) was added to the solid and then run through a biotage system (gradient elution, 5% to 40% v/v of EtOAc in hexane). The pale-yellow solid was isolated (60 mg, 16%). ^{1}H NMR (400 MHz, $CDCl_3$): δ 7.58 (dt, J = 8, 1.8 Hz, 4H), 7.24 (dt, J = 8, 1.8 Hz, 4H), 3.02 (s, 4H). ^{13}C{^{1}H} NMR (101 MHz, $CDCl_3$) δ 146.1 132.3, 129.3, 118.9, 110.3, 37.2.

1,1'-(1,2-Ethanediyl)bis[4-(trifluoromethyl)benzene]: In a nitrogen-filled glovebox, a 215 mM 4-(trifluoromethyl)benzyl bromide solution in anhydrous THF was prepared before the reactions. A snap vial with a stirring bar was charged with 20 mg (0.0375 mmol) of $(N\text{-}DMBI)_2$ and 3.65 mL of anhydrous THF. 4-(Trifluoromethyl)benzyl bromide (0.075 mmol) was added by pipetting an aliquot (348 µL) of the stock solution into the vial and resulting in a 4 mL solution ([RX] = 18.7 mM). The vial was taken out of the glovebox immediately after the mixing and subjected to UV light for 6 h in the HepatoChem photoreactor at room temperature. After the reaction, THF was removed by rotavap without heating. The dimer product (R_f = 0.8) was isolated by preparative TLC using an eluent of hexane/acetone 8:2 (v/v). The TLC plate solids were extracted with acetone-d_6, and 4.8 mg of 1,4-dimethoxybenzene was added to the solution for the determination of dimer yield. The mixture was found by ^{1}H NMR spectroscopy to give 1,1'-(1,2-ethanediyl)bis[4-

(trifluoromethyl)benzene] in 97% NMR yield. ^{1}H NMR (400 MHz, acetone-d_6) δ 7.64 (d, J = 8.0 Hz, 4H), 7.50 (d, J = 8.0 Hz, 4H), 3.11 (s, 4H). ^{19}F NMR (376 MHz, acetone-d_6) δ −62.80. ^{13}C{^{1}H} NMR (101 MHz, acetone-d_6) δ 146.1, 129.2, 127.7 (q, J_{CF} = 32 Hz), 125.1 (q, J_{CF} = 4.0 Hz), 124.6 (q, J_{CF} = 272 Hz), 36.8.

4. UV–vis spectra of benzyl bromide and dimers

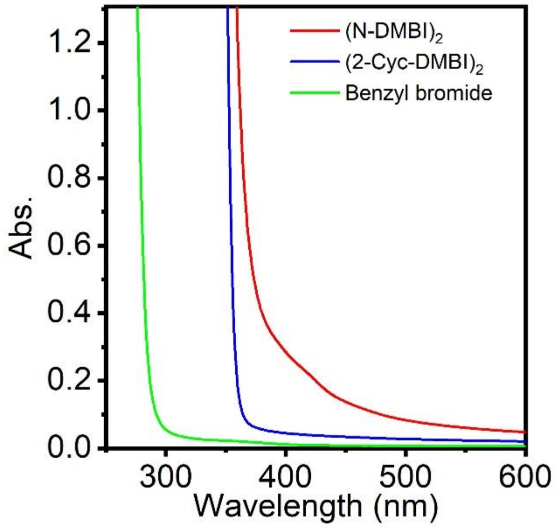

Figure S4. UV–vis spectra of the benzyl bromide (53 mM) in anhydrous THF. (N-DMBI)$_2$ and (2-Cyc-DMBI)$_2$ spectra in THF (1.9 mM and 2.2 mM, respectively) are stacked for comparison.

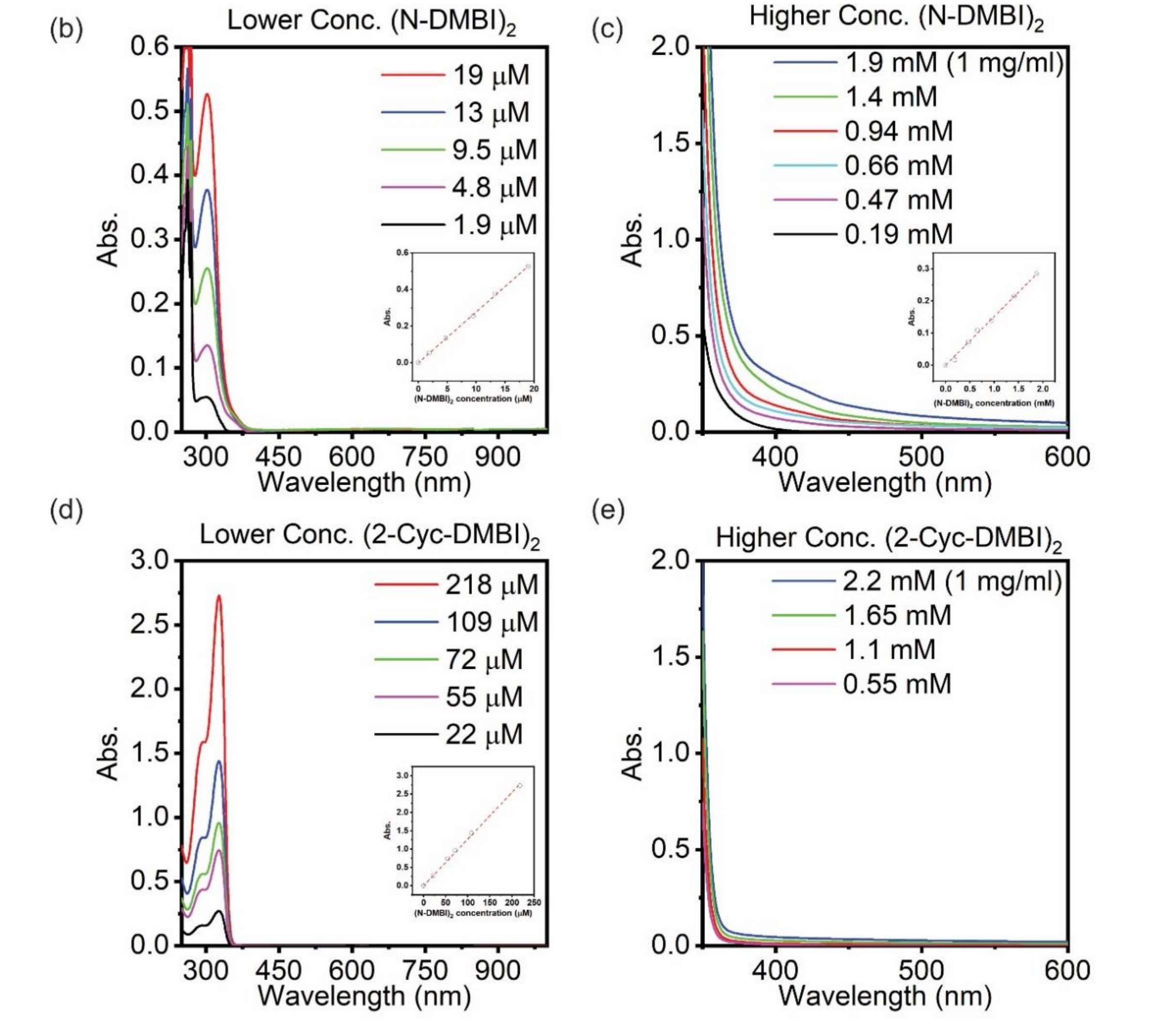

Figure S5. UV–vis spectra of the dimers in anhydrous THF at lower (μM) and higher (mM) concentration regimes with plots of absorbance vs. concentration at the peak wavelengths in (a) and (b), or in (c) at 400 nm, used to determine absorptivities. The linear fit in (c) suggests the low energy shoulder is a molecular feature (as also suggested by the TD-DFT calculations and by its occurance in different solvents; see Figure S6) rather than an aggregation-related feature.

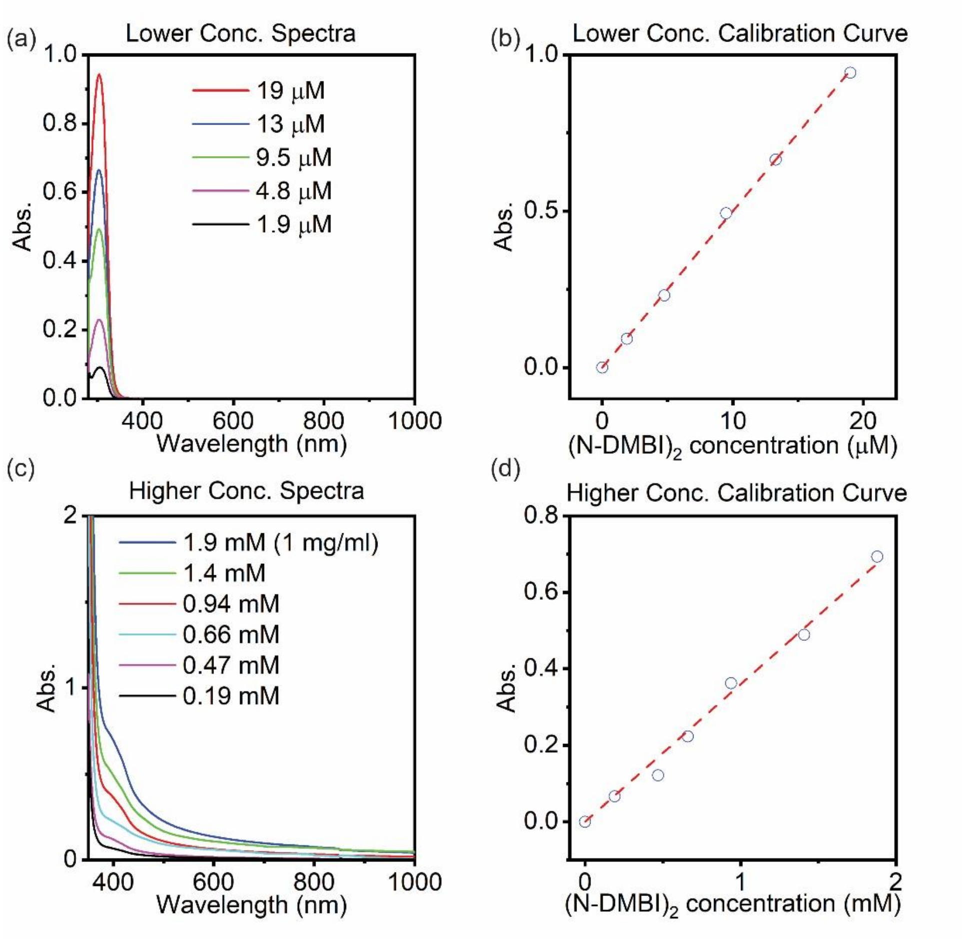

Figure S6. UV–vis spectra of the dimers in anhydrous toluene at lower (μM) and higher (mM) concentration regimes with plots of absorbance vs. concentration at the peak wavelengths in (a) and (b), or in (c) at 400 nm, used to determine absorptivities.

5. Cyclic voltammograms of organic halides

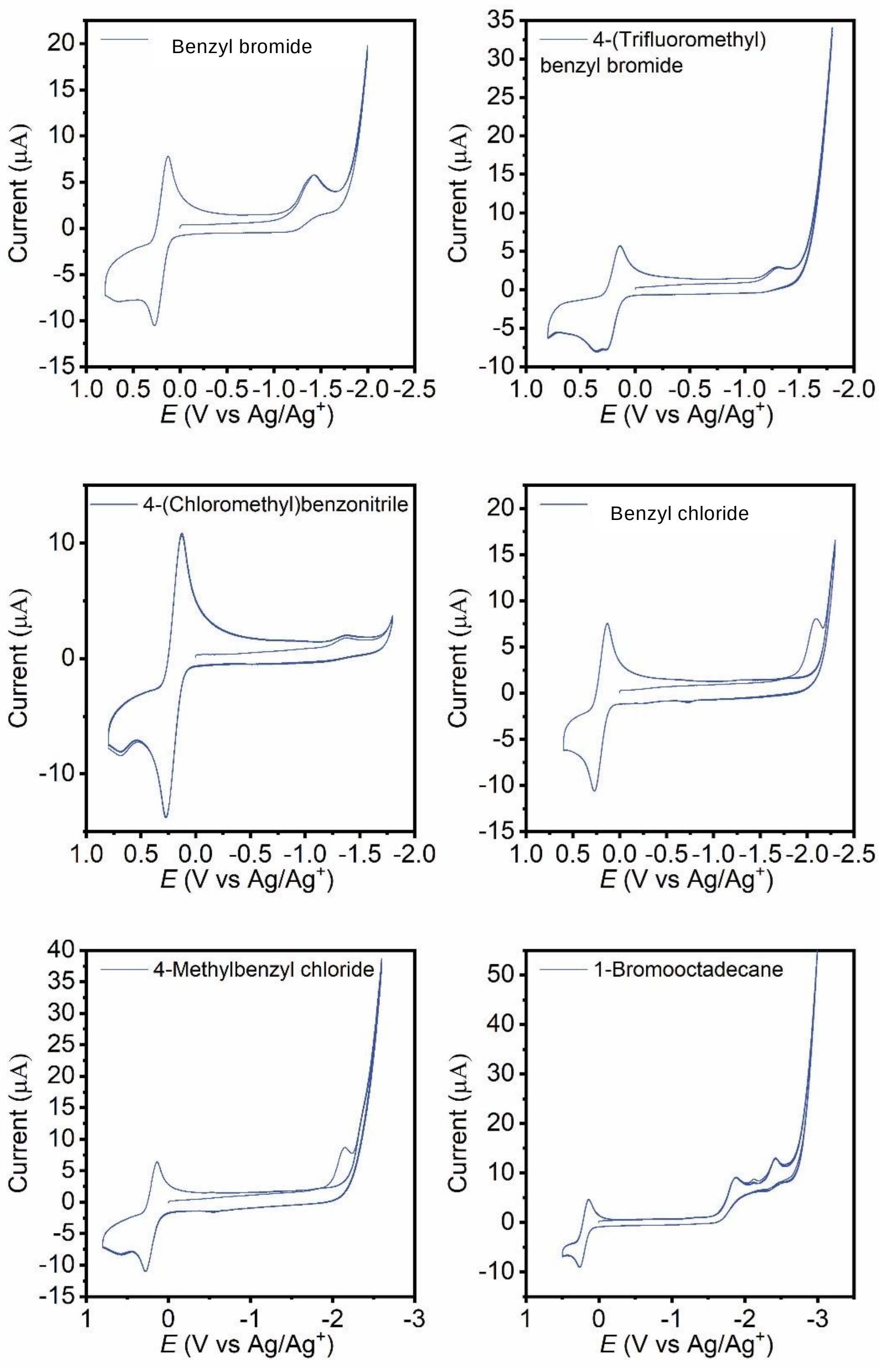

Figure S7. Cyclic voltammograms of benzyl or alkyl halides (10 mM) under N_2. Conditions: scan rate of 100 mV s^{-1}, N_2 sparged anhydrous THF solvent, 0.1 M Bu_4NPF_6, internal $FeCp_2$ (responsible for the reversible feature at positive potential and subsequently used to obtain the values referenced to $FeCp_2^{+/0}$ reported in Table 2).

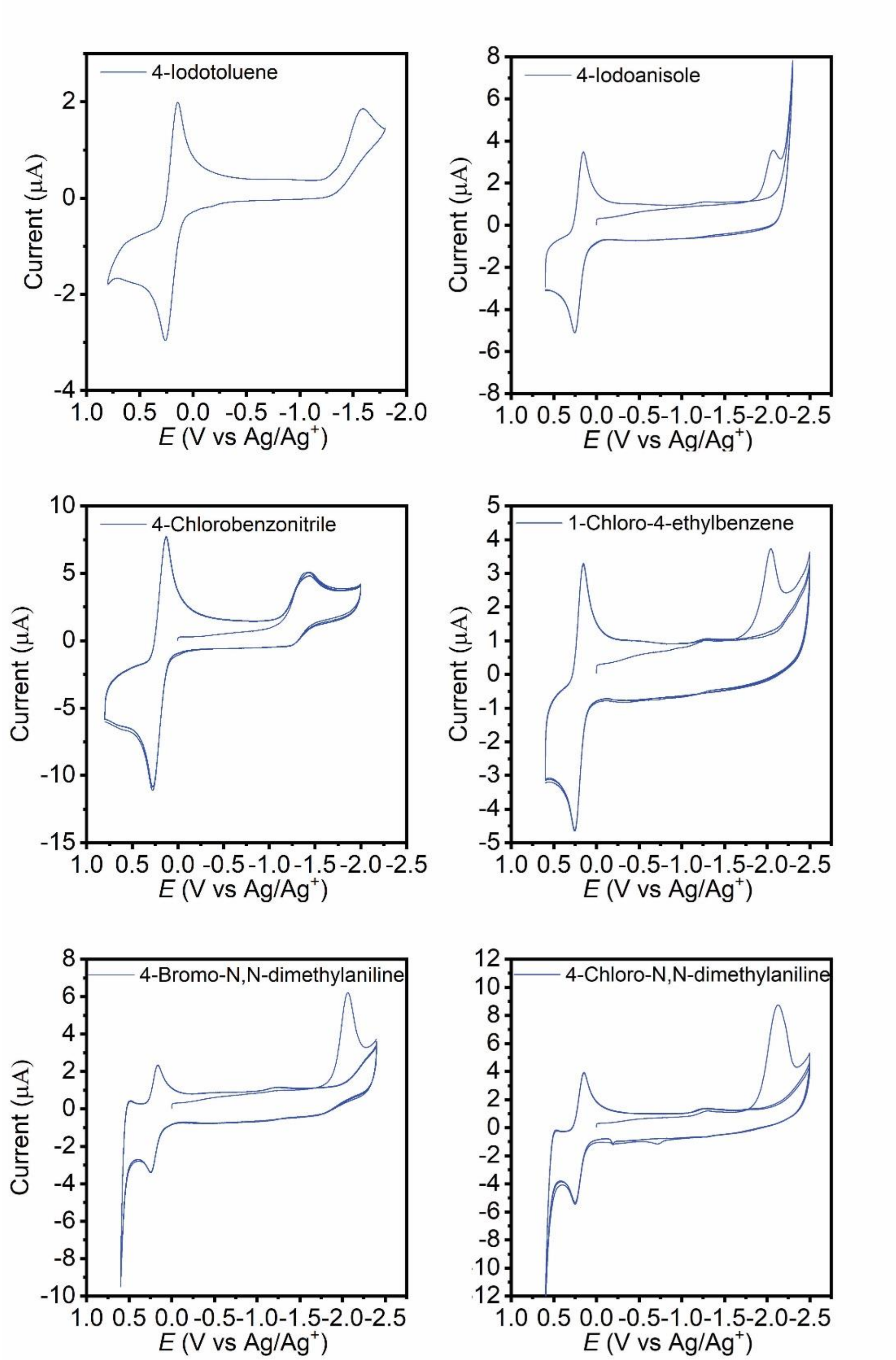

Figure S8. Cyclic voltammograms of aryl halides (10 mM) under N_2. Conditions: scan rate of 100 mV s^{-1}, N_2 sparged anhydrous THF solvent, 0.1 M Bu_4NPF_6, internal $FeCp_2$ (responsible for the reversible feature at positive potential and subsequently used to obtain the values referenced to $FeCp_2^{+/0}$ reported in Table 2).

6. NMR crossover experiments

We performed NMR crossover experiments as a possible means of confirming photoinduced dimer cleavage. Specifically, we exposed a mixture of $(N\text{-}DMBI)_2$ and $(Cyc\text{-}DMBI)_2$ in benzene-d_6 to 365 nm light and used ^1H Nuclear Overhauser effect (NOE) experiments to examine whether the mixed dimer was formed. However, no evidence for the formation of such a dimer was obtained. Specifically, no evidence for NOE interactions between Cyc- and N-DMBI monomers was observed. For example, when i-H (2H, 1.72 ppm) of $(Cyc\text{-}DMBI)_2$ monomer is irradiated, identical NOE that is from the α-CH_3 of $(Cyc\text{-}DMBI)_2$ (6H, 2.65 ppm) is seen (Figure b), regardless of UV exposure. While this finding does not provide evidence for photoinduced dissociation it does not rule it out. It is also possible that: (i) one dimer dissociates and the other doesn't; (ii) both dimers dissociate, but redimerize selectively; or (iii) both dimers dissociate but one or both monomer species redimerize before they can escape their solvent cage.

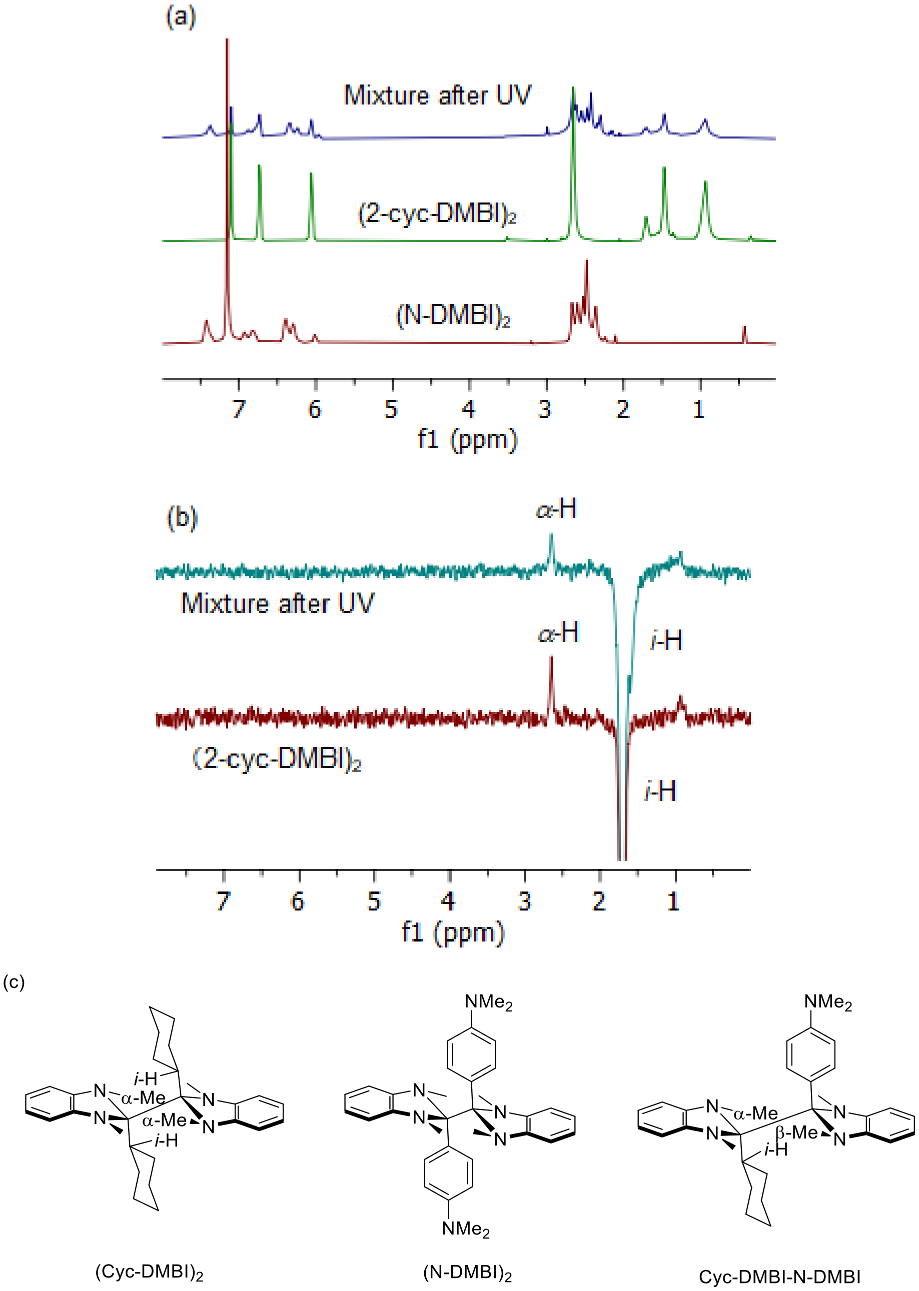

Figure S9. (a) ^{1}H NMR spectra of pure (2-Cyc-DMBI)$_2$, (N-DMBI)$_2$, and the mixture of two dimers ([(N-DMBI)$_2$]/[(2-Cyc-DMBI)$_2$] 1:1, 10.9 mM) after exposure to UV (365 nm) for 0.5 h in benzene-d_6 (b) 1D NOE spectra of pure (2-Cyc-DMBI)$_2$ and the mixture of two dimers [(N-DMBI)$_2$]/[(2-Cyc-DMBI)$_2$] 1:1, 10.9 mM) after exposure to UV for 0.5 h in benzene-d_6. (c) The structures of the (2-Cyc-DMBI)$_2$, (N-DMBI)$_2$, and the hypothetical crossover product 2-Cyc-DMBI-N-DMBI. Protons that are relevant in the observed NOEs in (b) are labeled in the structures.

7. TD-DFT calculations

The DFT calculations were performed using the Minnesota 06 (M06) functional with the 6-31G(d,p) basis set. The geometry of each species was optimized and the subsequently used as the basis for time-dependent DFT calculations to obtain excitation energies and oscillator strengths. An artificial broadening of σ = 0.3 was applied to each peak to produce the simulated optical absorption spectra shown. To better understand the nature of the excited states, natural transition orbitals (NTO) were calculated for select species. All calculations were performed using the Gaussian16 Rev. A.03 quantum chemical calculation package [4].

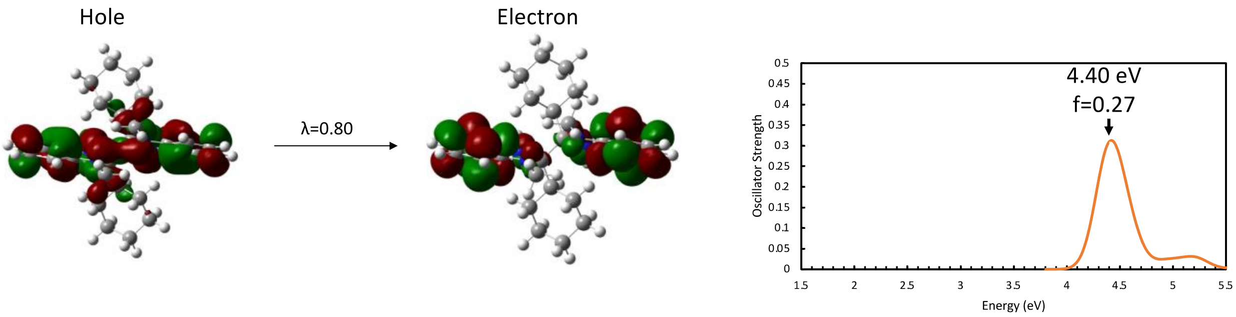

Figure S10. NTOs for the S_0–S_1 transition of $(\text{Cyc-DMBI})_2$ shown alongside a simulated absorption spectrum.

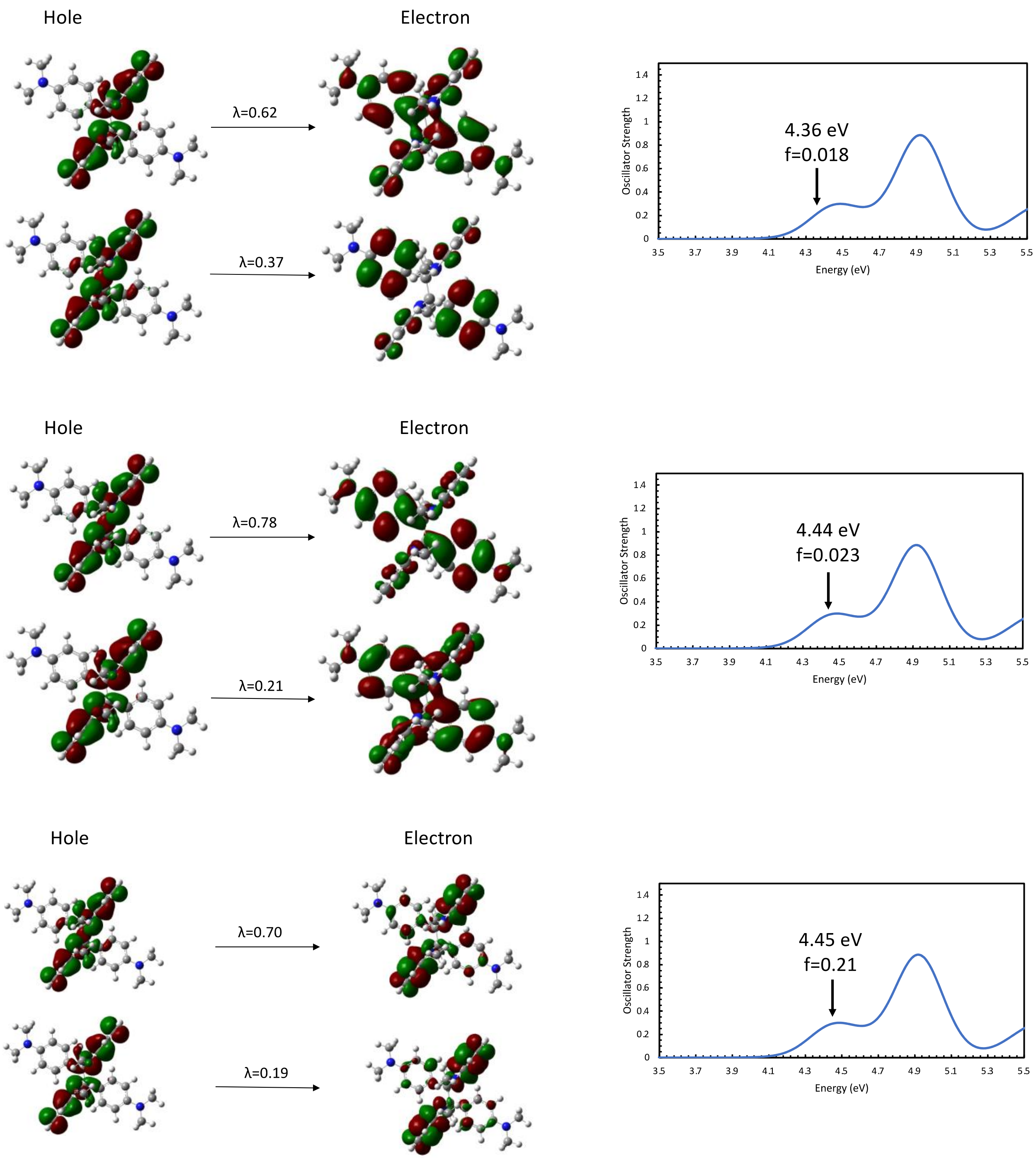

Figure S11. From top to bottom: NTOs for the S_0–S_4, S_0–S_5, and S_0–S_6 transitions of $(N\text{-DMBI})_2$ with the oscillator strengths and transition energies indicated on a simulated absorption spectrum.

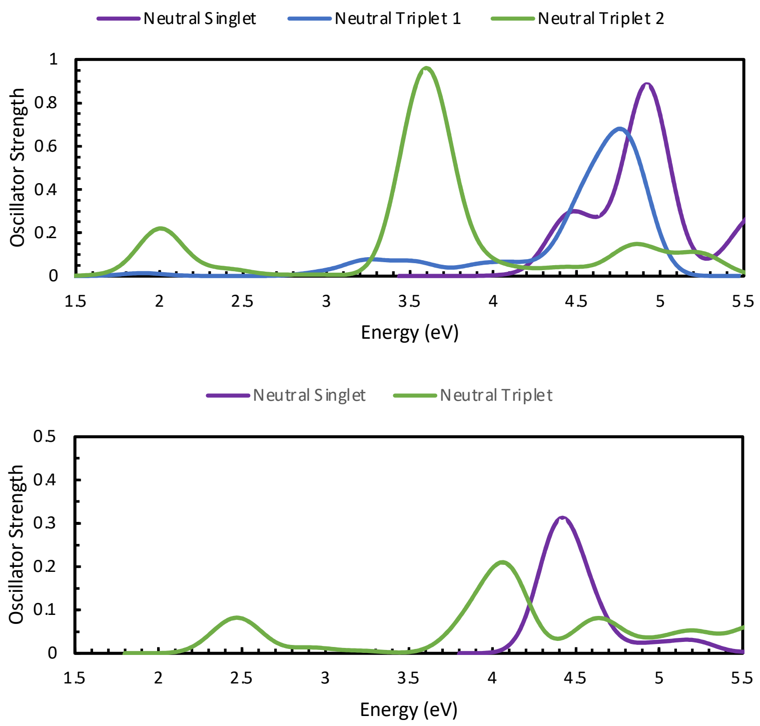

Figure S12. Top and bottom: simulated absorption spectra for S_0 and T_1 states of the $(N\text{-}DMBI)_2$ and $(Cyc\text{-}DMBI)_2$, respectively. Note that triplet 1 for $(N\text{-}DMBI)_2$ corresponds to a structure minimized from one of the molecules in the crystal structure [5]. However, this structure has imaginary vibrational frequency indicating that it is in a local energy minimum. Triplet 2 is calculated at the true minima (and ca. 3 eV lower in energy).

8. Transient absorption spectroscopy

Transient absorption data were collected using the output from a 1 kHz Ti:Sapphire regenerative amplifier (Coherent Libra) with an 800 nm fundamental and a 100 fs pulse width. The 350 nm pump was generated in an optical parametric amplifer (TOPAS-C, Light Conversion) and the probe was created via supercontinuum generation by focusing the beam into a thin sapphire window (λ_{probe} = 440–800 nm). The pump and probe were spatially overlapped at the sample and the probe was delayed relative to the pump using a mechanical delay stage that allows time delays from −2 ps through 5 ns. The pump was chopped at 500 Hz to modulate the pump on and off. The change in absorbance was measured using a fiber optic coupled multichannel spectrometer with a CMOS sensor. Data were collected and analyzed using Helios and Surface xplorer (Ultrafast Systems). The data were chirp corrected.

9. NMR Spectra

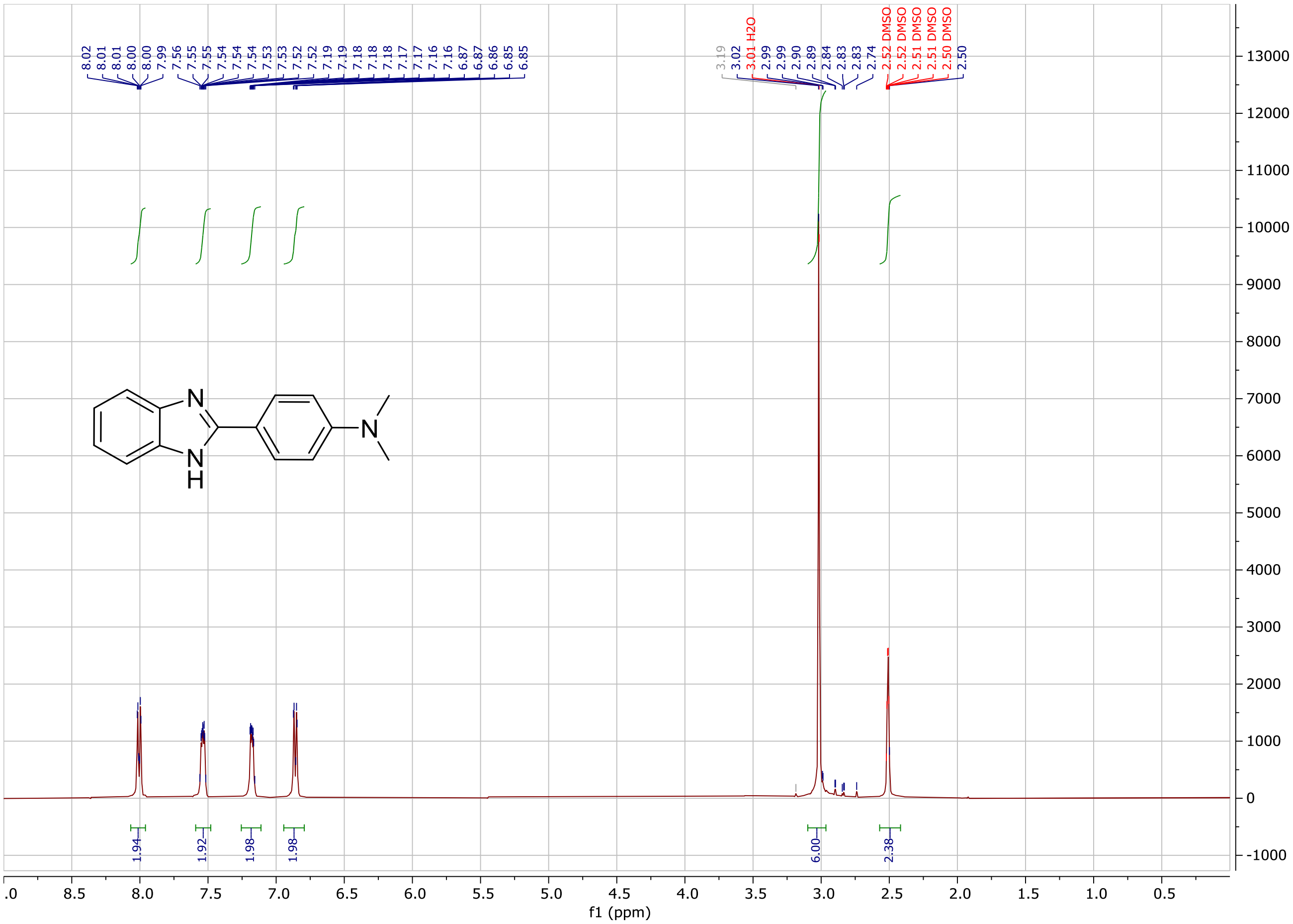

Figure S13. ^{1}H NMR spectrum of (400 MHz, DMSO-d_6) of 4-(1*H*-benzimidazol-2-yl)-*N*,*N*-dimethylaniline.

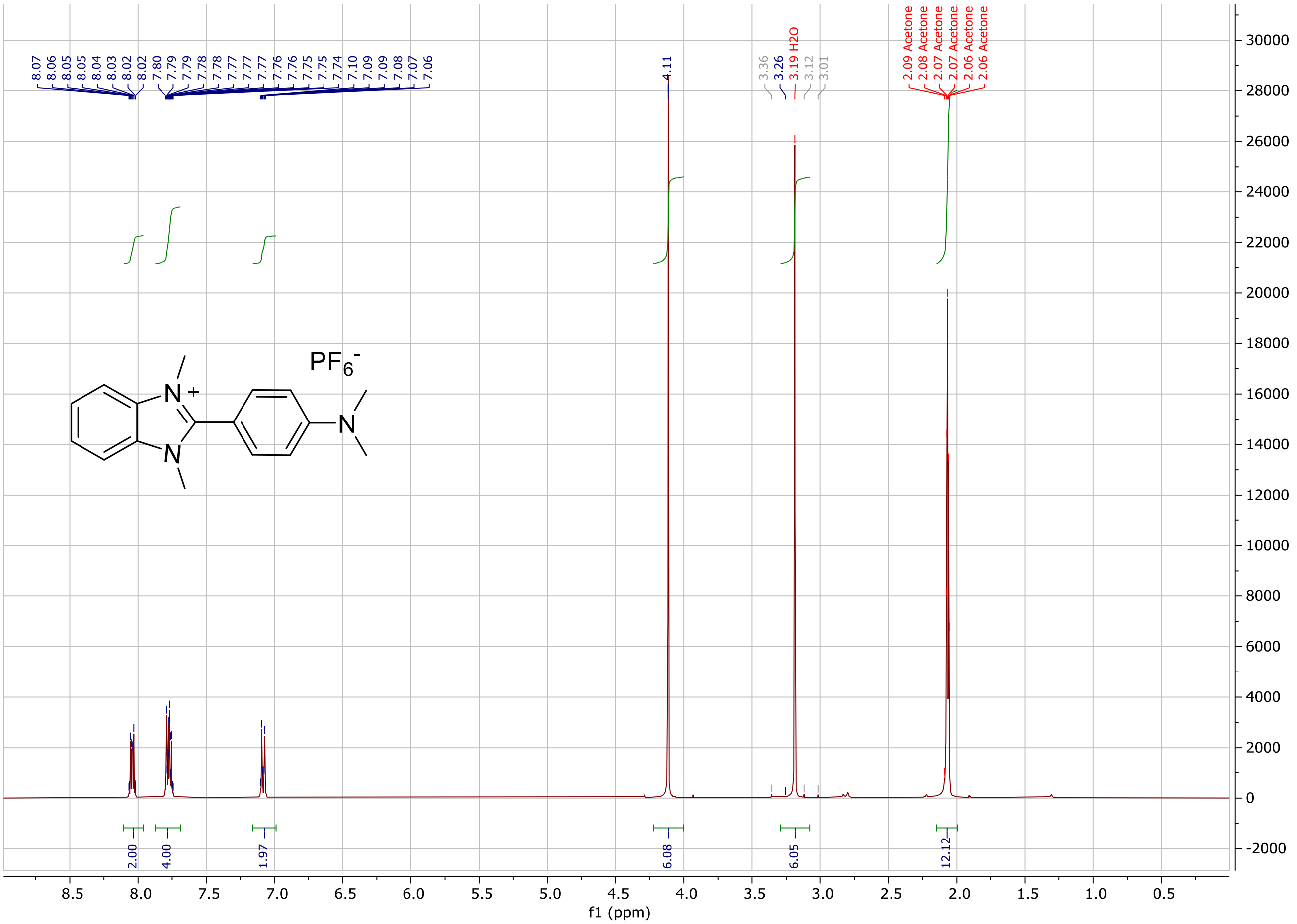

Figure S14. ^{1}H NMR spectrum (400 MHz, acetone-d_6) of N-DMBI^{+}PF$_6^{-}$.

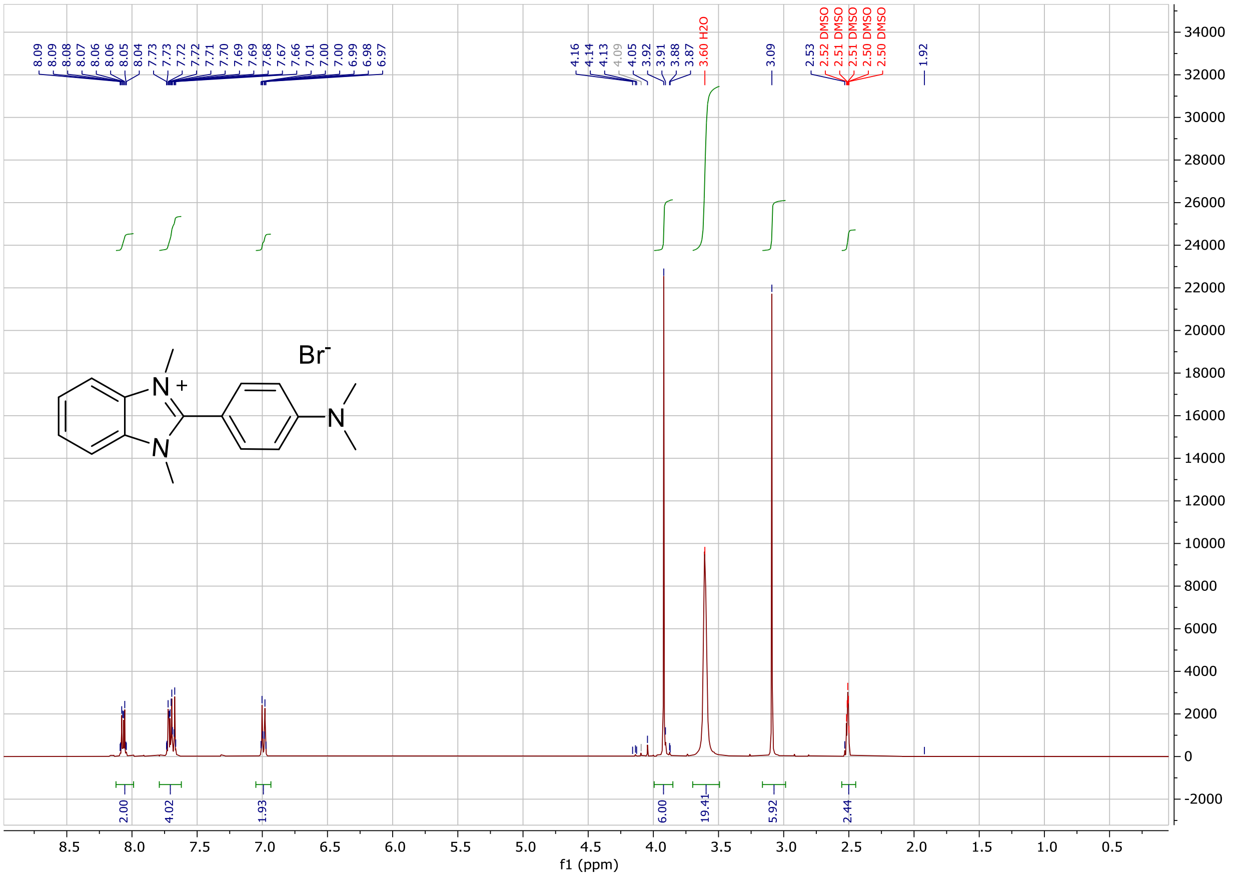

Figure S15. ^{1}H NMR spectrum (400 MHz, DMSO-d_6) of N-DMBI^{+}Br$^{−}$ formed during the dehalogenation of benzyl bromide (18 h reaction).

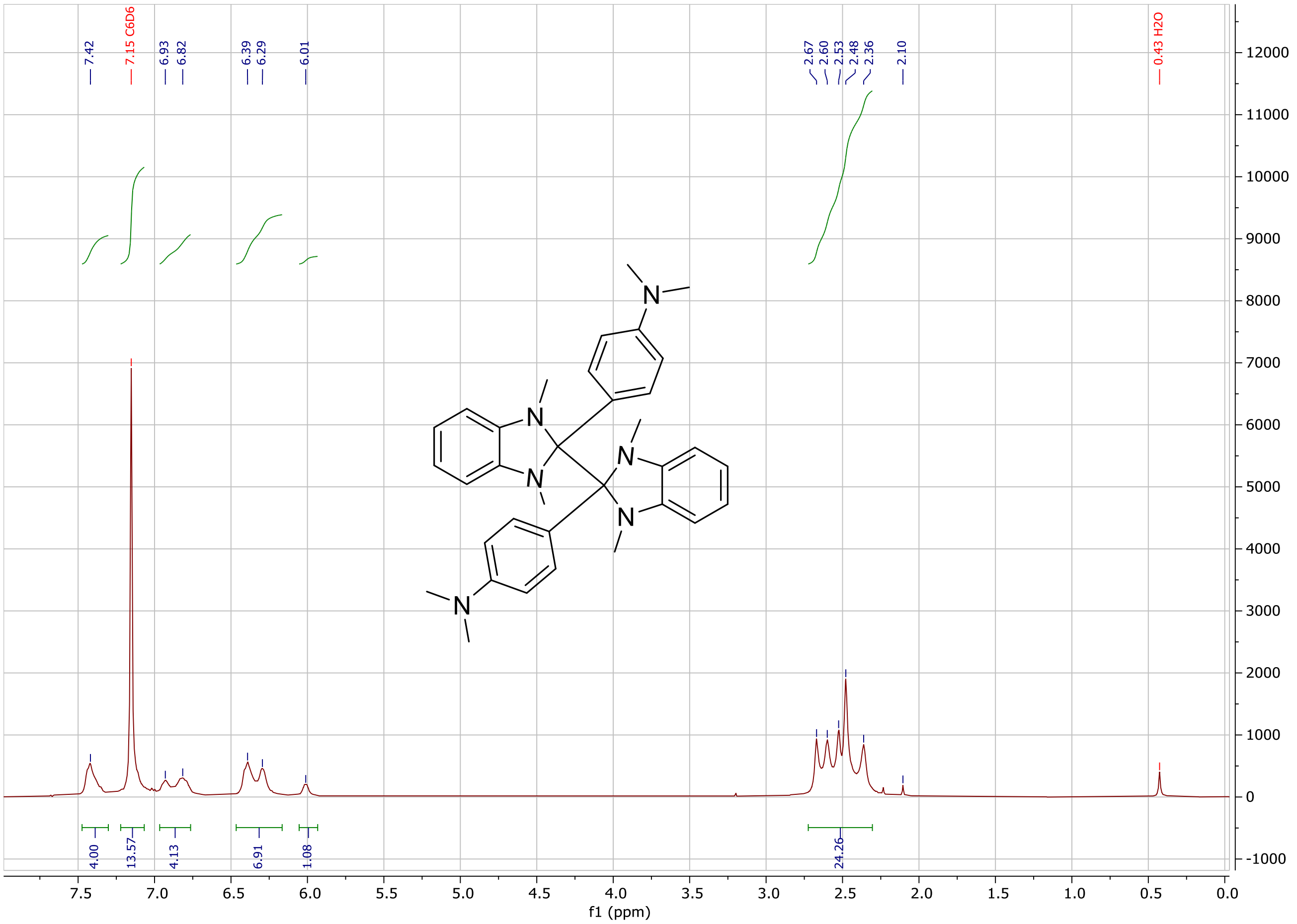

Figure S16 ^{1}H NMR spectrum (400 MHz, C_6D_6) of (N-DMBI)$_2$.

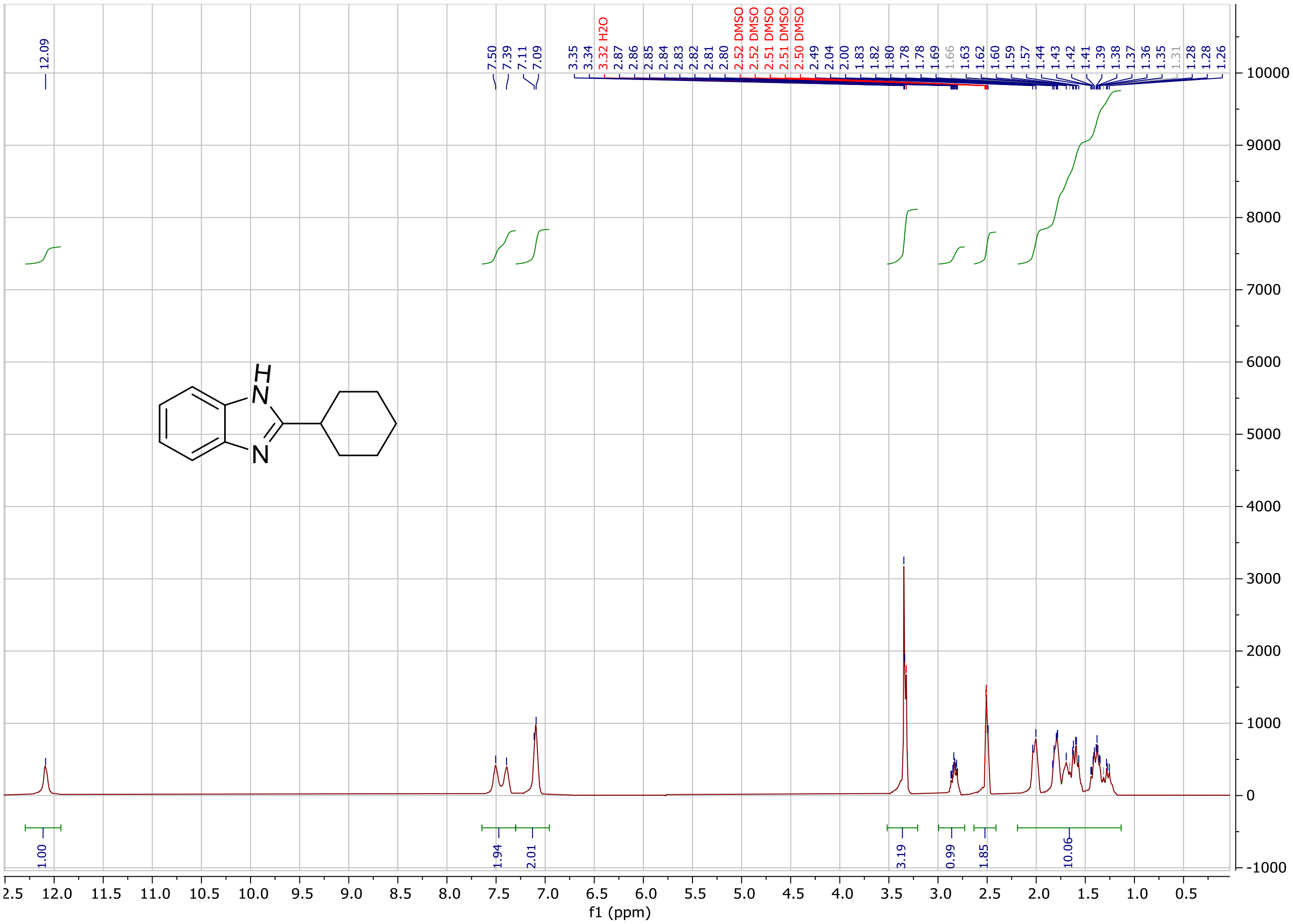

Figure S17. ^{1}H NMR spectrum (400 MHz, DMSO-d_6) of 4-(1*H*-benzimidazol-2-yl)-cyclohexane.

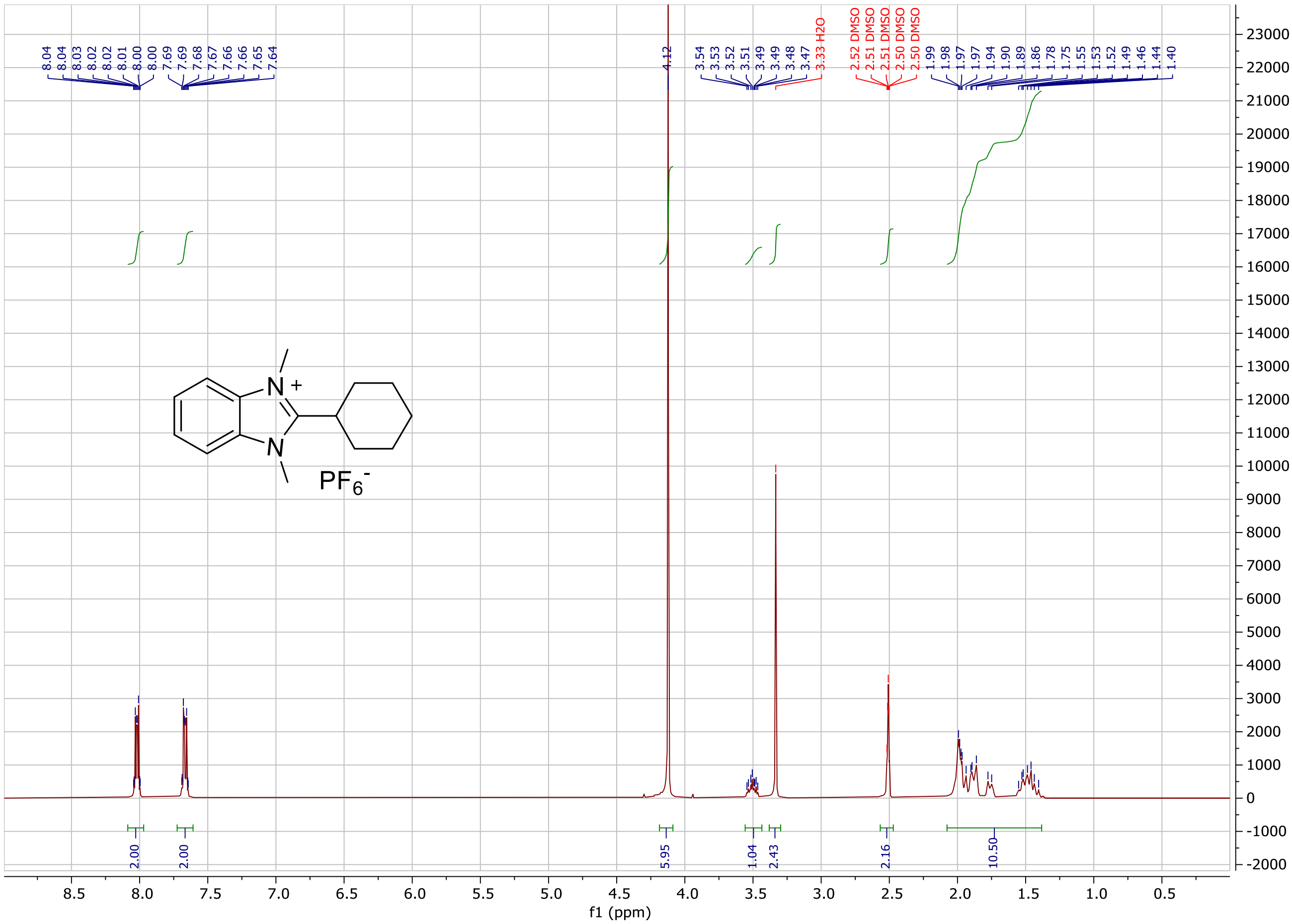

Figure S18. ^{1}H NMR spectrum (400 MHz, DMSO-d_6) of 2-Cyc-DMBI^{+}PF$_6^{-}$.

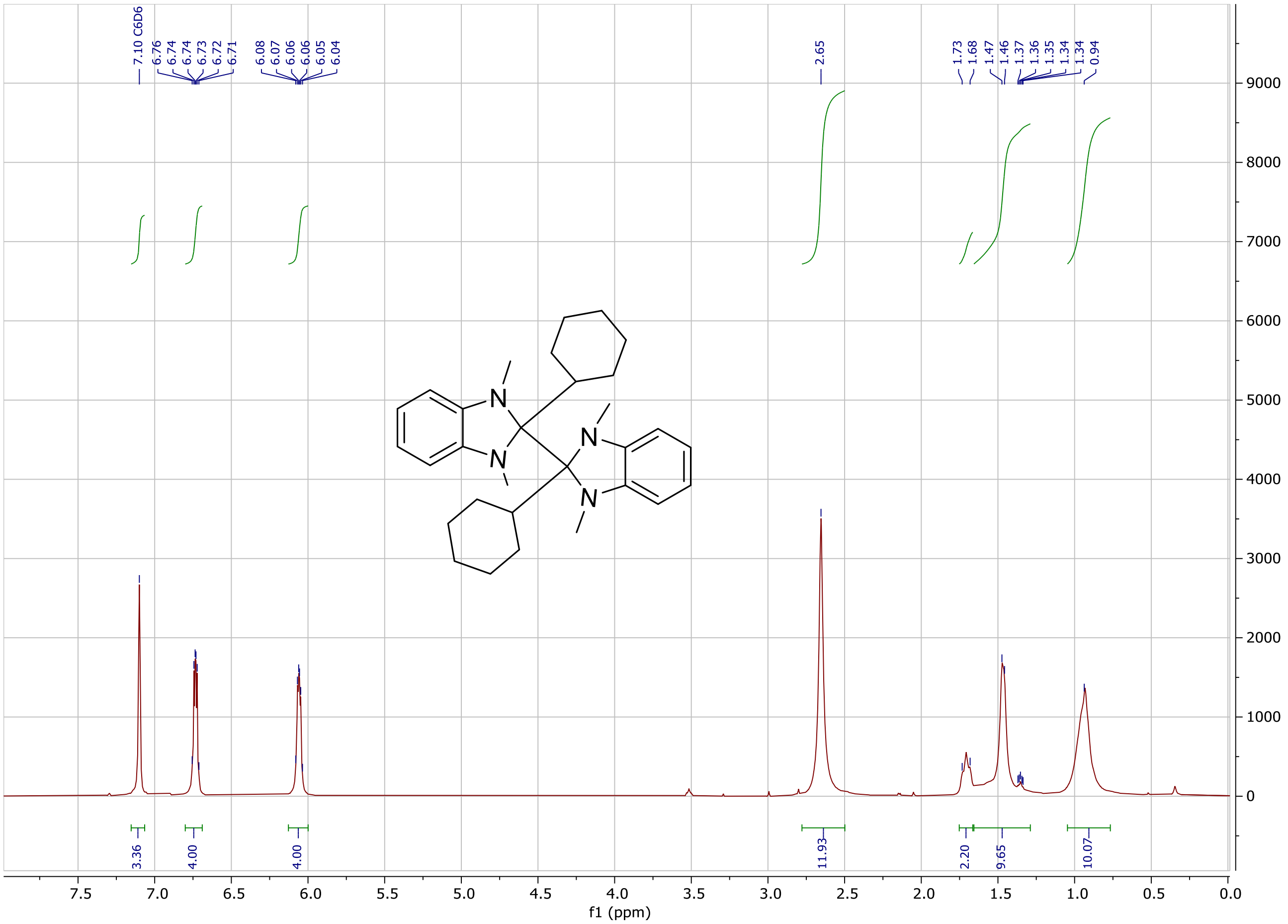

Figure S19. ^{1}H NMR spectrum (400 MHz, C_6D_6) of (2-Cyc-DMBI)$_2$.

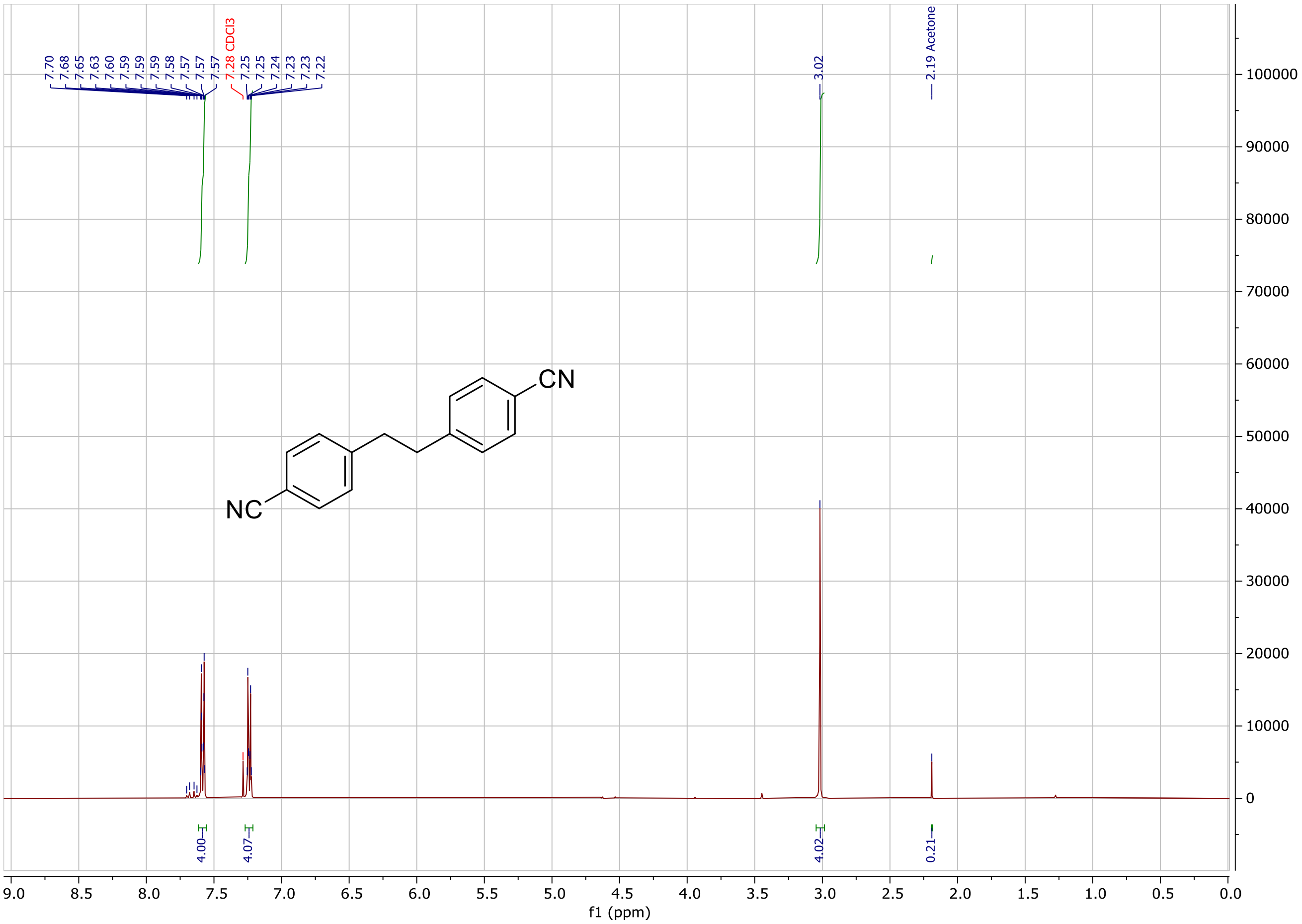

Figure S20. ^{1}H NMR spectrum (400 MHz, $CDCl_3$) of 4,4'-(1,2-ethanediyl)dibenzonitrile.

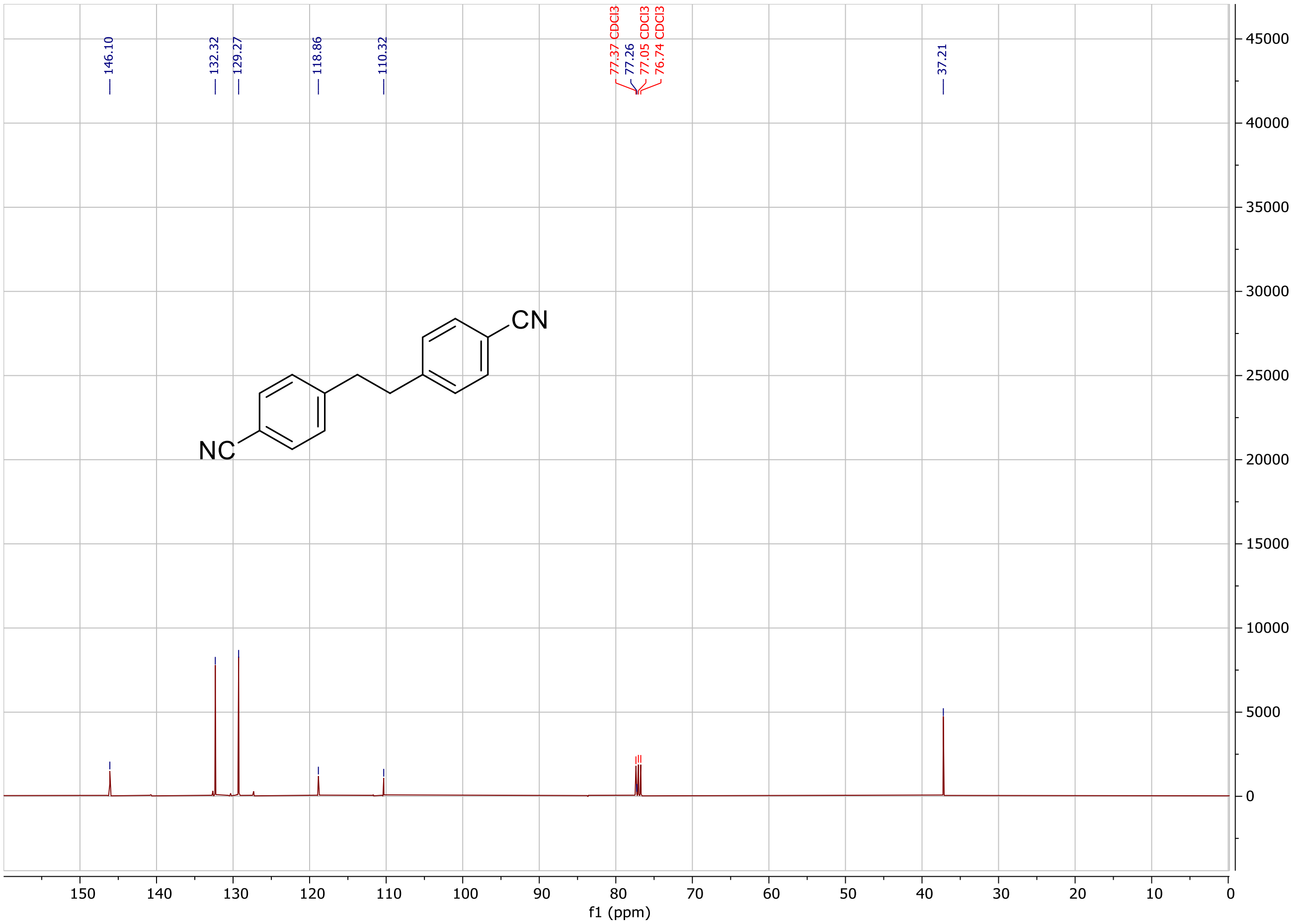

Figure S21. $^{13}C\{^1H\}$ NMR spectrum (101 MHz, $CDCl_3$) of 4,4'-(1,2-ethanediyl)dibenzonitrile.

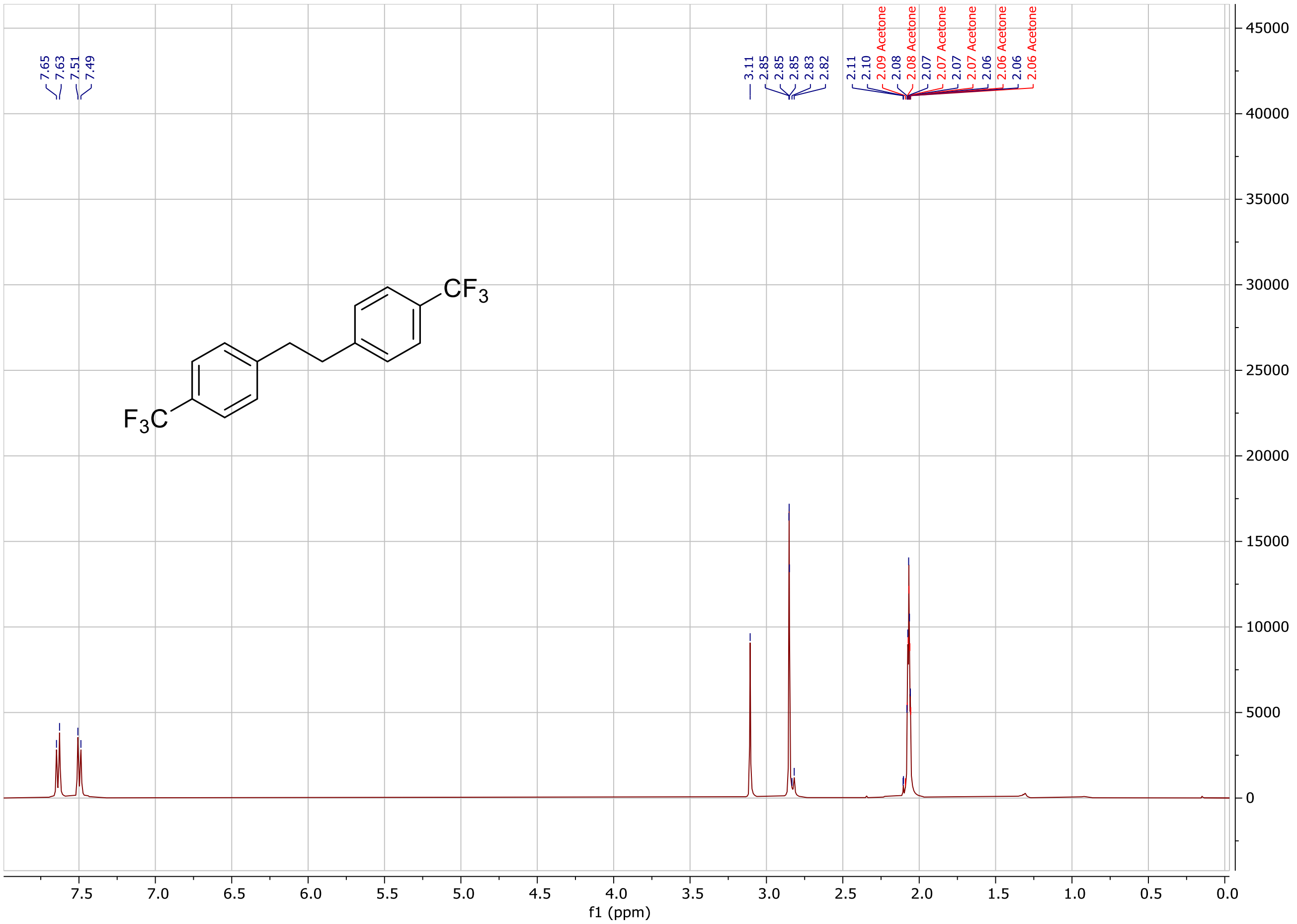

Figure S22. ^{1}H NMR spectrum (400 MHz, acetone-d_6) of 1,1'-(1,2-ethanediyl)bis[4-(trifluoromethyl)benzene].

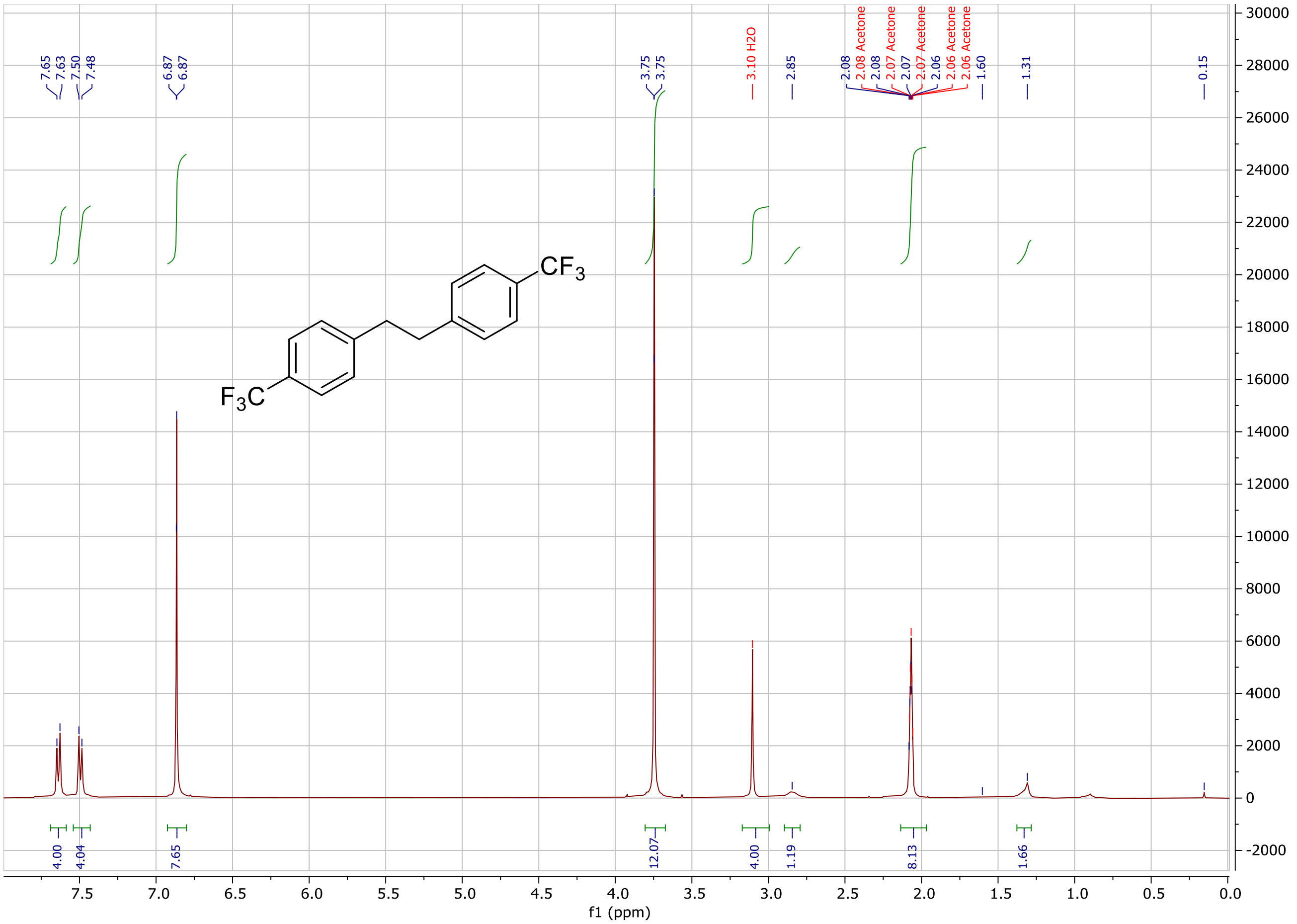

Figure S23. ^{1}H NMR spectrum (400 MHz, acetone-d_6) of 1,1'-(1,2-ethanediyl)bis[4-(trifluoromethyl)benzene] with internal standard.

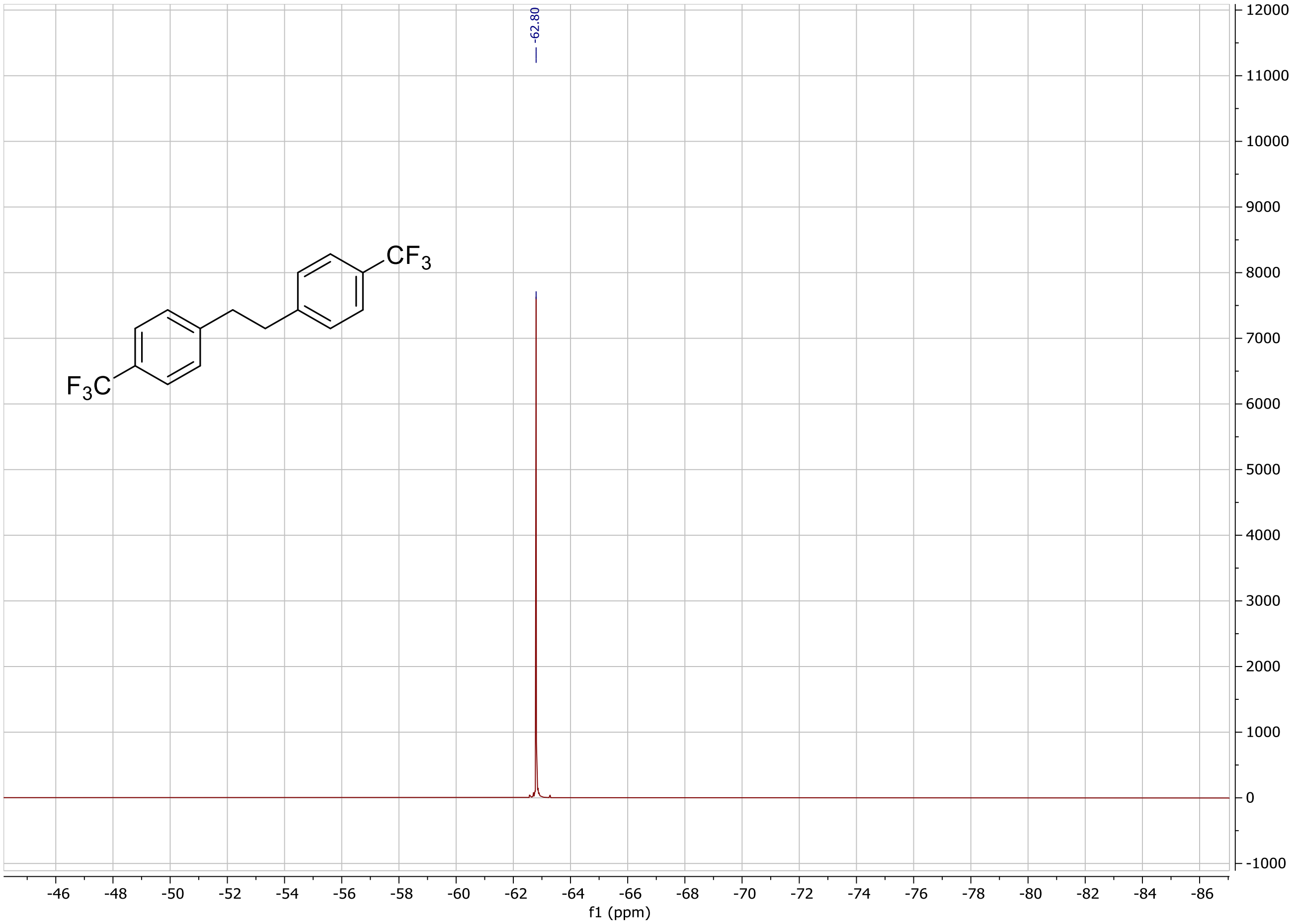

Figure S24. ^{19}F NMR spectrum (376 MHz, acetone-d_6) of 1,1'-(1,2-ethanediyl)bis[4-(trifluoromethyl)benzene].

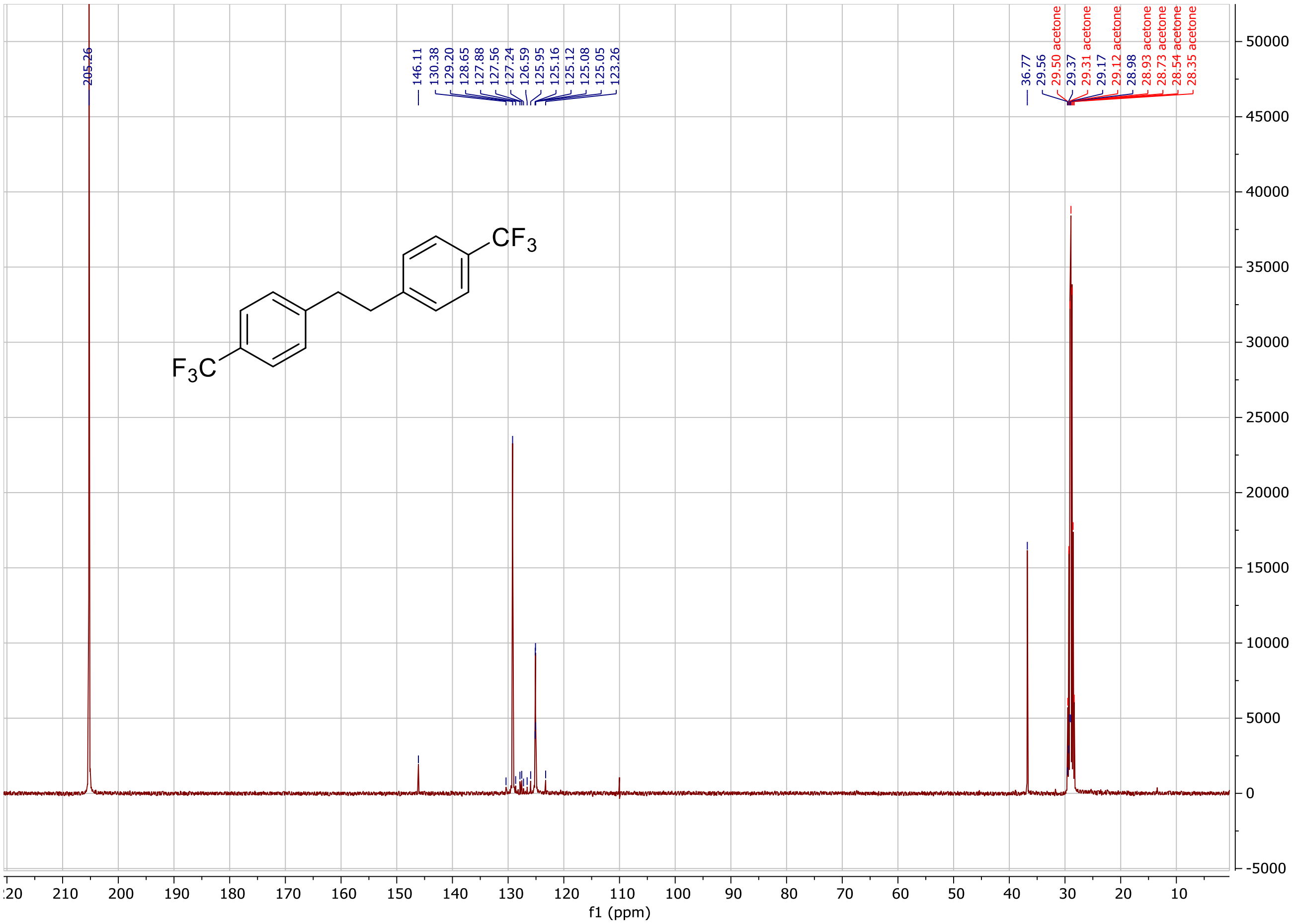

Figure S24. $^{13}C\{^1H\}$ NMR (101 MHz, acetone-d_6) of 1,1'-(1,2-ethanediyl)bis[4-(trifluoromethyl)benzene].

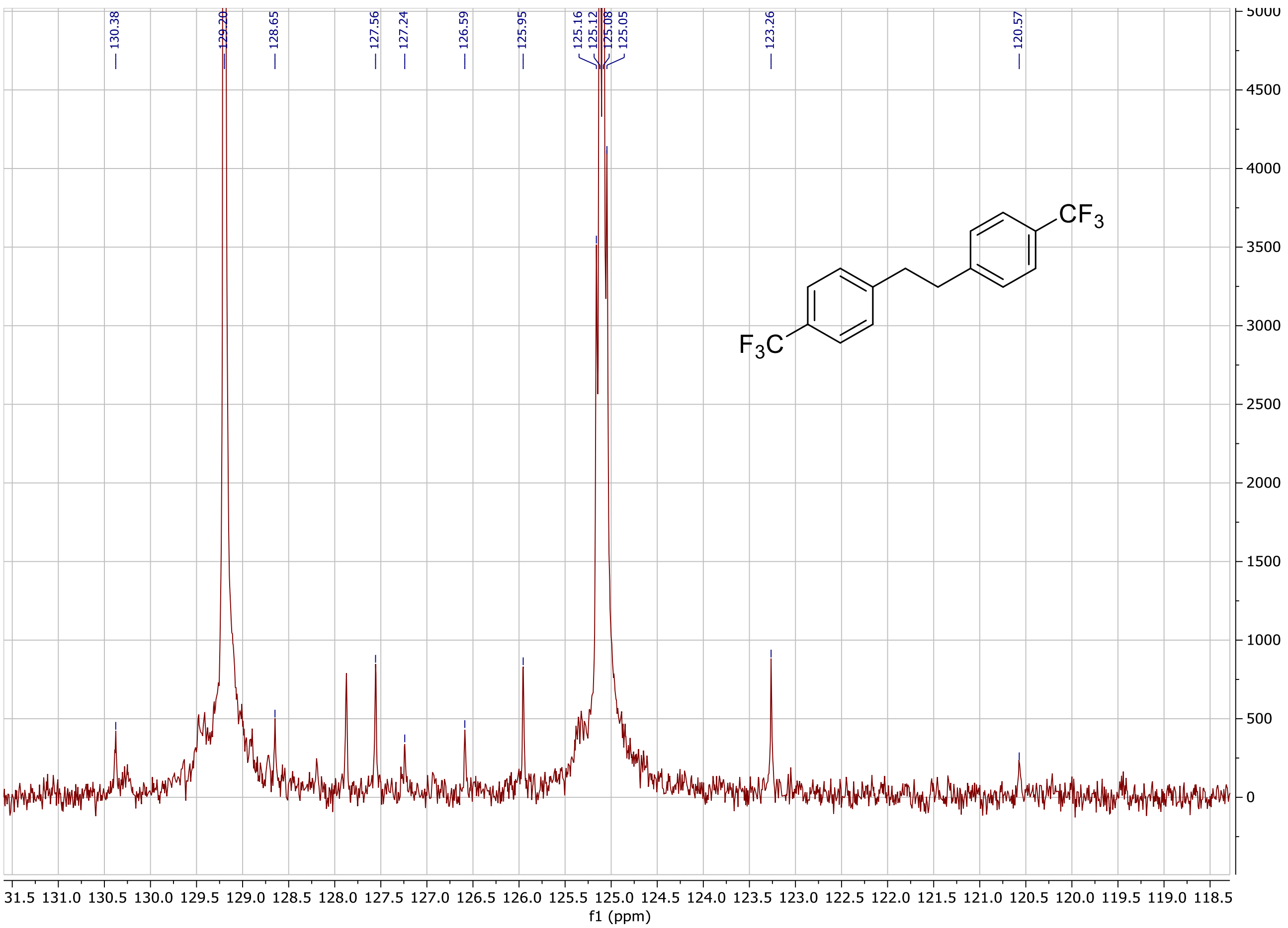

Figure S25. Expanded $^{13}C\{^1H\}$ NMR spectrum (101 MHz, acetone-d_6) of 1,1'-(1,2-ethanediyl)bis[4-(trifluoromethyl)benzene.

References

1. Un, H.-I.; Gregory, S. A.; Mohapatra, S. K.; Xiong, M.; Longhi, E.; Lu, Y.; Rigin, S.; Jhulki, S.; Yang, C.-Y.; Timofeeva, T. V.; Wang, J.-Y.; Yee, S. K.; Barlow, S.; Marder, S. R.; Pei, J. *Adv. Energy Mater.* **2019**, *9*, 1900817. doi: 10.1002/aenm.201900817
2. Naab, B. D.; Gu, X.; Kurosawa, T.; To, J. W. F.; Salleo, A.; Bao, Z. *Adv. Electron. Mater.* **2016**, *2*, 1600004. doi: 10.1002/aelm.201600004
3. Yu, D.; To, W.-P.; Tong, G. S. M.; Wu, L.-L.; Chan, K.-T.; Du, L.; Phillips, D. L.; Liu, Y.; Che, C.-M. *Chem. Sci.* **2020**, *11*, 6370-6382. doi: 10.1039/d0sc01340d
4. *Gaussian 16, Revision A.03*, Frisch, M. J.; Trucks, G. W.; Schlegel, H. B.; Scuseria, G. E.; Robb, M. A.; Cheeseman, J. R.; Scalmani, G.; Barone, V.; Petersson, G. A.; Nakatsuji, H.; Li, X.; Caricato, M.; Marenich, A. V.; Bloino, J.; Janesko, B. G.; Gomperts, R.; Mennucci, B.; Hratchian, H. P.; Ortiz, J. V.; Izmaylov, A. F.; Sonnenberg, J. L.; Williams-Young, D.; Ding, F.; Lipparini, F.; Egidi, F.; Goings, J.; Peng, B.; Petrone, A.; Henderson, T.; Ranasinghe, D.; Zakrzewski, V. G.; Gao, J.; Rega, N.; Zheng, G.; Liang, W.; Hada, M.; Ehara, M.; Toyota, K.; Fukuda, R.; Hasegawa, J.; Ishida, M.; Nakajima, T.; Honda, Y.; Kitao, O.; Nakai, H.; Vreven, T.; Throssell, K.; Montgomery, J. A., Jr.; Peralta, J. E.; Ogliaro, F.; Bearpark, M. J.; Heyd, J. J.; Brothers, E. N.; Kudin, K. N.; Staroverov, V. N.; Keith, T. A.; Kobayashi, R.; Normand, J.; Raghavachari, K.; Rendell, A. P.; Burant, J. C.; Iyengar, S. S.; Tomasi, J.; Cossi, M.; Millam, J. M.; Klene, M.; Adamo, C.; Cammi, R.; Ochterski, J. W.; Martin, R. L.; Morokuma, K.; Farkas, O.; Foresman, J. B.; Fox, D. J. Gaussian, Inc., Wallingford CT, 2016.
5. Mohapatra, S. K.; Al Kurdi, K.; Jhulki, S.; Bogdanov, G.; Bacsa, J.; Conte, M.; Timofeeva, T. V.; Marder, S. R.; Barlow, S. *Beilstein J. Org. Chem*. **2023**, *19*, 1651-1663. doi: 10.3762/bjoc.19.121
